# JContextExplorer: a tree-based approach to facilitate cross-species genomic context comparison

**DOI:** 10.1186/1471-2105-14-18

**Published:** 2013-01-16

**Authors:** Phillip Seitzer, Tu Anh Huynh, Marc T Facciotti

**Affiliations:** 1 Department of Biomedical Engineering, One Shields Ave, University of California, Davis, CA, 95616, USA; 2 Genome Center, One Shields Ave, University of California, Davis, CA, 95616, USA; 3 Microbiology Graduate Group, One Shields Ave, University of California, Davis, CA, 95616, USA

**Keywords:** Genomic context, Genomic neighborhood, Comparative genomics, Java, GUI

## Abstract

**Background:**

Cross-species comparisons of gene neighborhoods (also called genomic contexts) in microbes may provide insight into determining functionally related or co-regulated sets of genes, suggest annotations of previously un-annotated genes, and help to identify horizontal gene transfer events across microbial species. Existing tools to investigate genomic contexts, however, lack features for dynamically comparing and exploring genomic regions from multiple species. As DNA sequencing technologies improve and the number of whole sequenced microbial genomes increases, a user-friendly genome context comparison platform designed for use by a broad range of users promises to satisfy a growing need in the biological community.

**Results:**

Here we present JContextExplorer: a tool that organizes genomic contexts into branching diagrams. We implement several alternative context-comparison and tree rendering algorithms, and allow for easy transitioning between different clustering algorithms. To facilitate genomic context analysis, our tool implements GUI features, such as text search filtering, point-and-click interrogation of individual contexts, and genomic visualization via a multi-genome browser. We demonstrate a use case of our tool by attempting to resolve annotation ambiguities between two highly homologous yet functionally distinct genes in a set of 22 alpha and gamma proteobacteria.

**Conclusions:**

JContextExplorer should enable a broad range of users to analyze and explore genomic contexts. The program has been tested on Windows, Mac, and Linux operating systems, and is implemented both as an executable JAR file and java WebStart. Program executables, source code, and documentation is available at http://www.bme.ucdavis.edu/facciotti/resources_data/software/.

## Background

As genomic sequencing becomes increasingly accurate, cheaper, and widespread, the need for tools to meaningfully interpret whole-organism genomic sequence data has increased. While a large collection of tools are devoted to sequence homology and phylogenetic analyses [1,2], far less attention has been paid to tools designed to meaningfully compare gene neighborhoods, or genomic contexts, across species. Differences among genomic contexts across species may indicate changes in the organization of functional transcription units [3,4], which ultimately result in differences among gene regulatory networks [5]. Genomic context may also be helpful in elucidating details of horizontal gene transfer and duplication events [6-8], and has been used to improve upon sequence-based gene annotation algorithms [9-11] and aid in the construction of protein-protein association networks [12]. In each of these investigations, a new method was created to meaningfully define and compare genomic contexts. The existence of a fast, accurate, user-friendly context comparison tool could have aided these investigations, and could encourage future researchers to incorporate genomic context analyses into their investigations.

In plant and animal species, a number of tools interrogating synteny (the degree that genes remain on corresponding chromosomes) and collinearity (the degree that genes remain on corresponding chromosomes and in order) [13] have been developed, such as MCScanX [14] and i-ADHoRe [15]). The Ensembl project [16] also utilizes syntenic data. These tools have many useful features, however lack a powerful visualization methods. Additionally, they do not focus on microbial species. In general, genomic context comparison methods applied to microbial species [3-11] have been highly customized, non-GUI based, and not readily extendable to other investigations. However, a number of rudimentary GUI platforms for exploration of annotated microbial genomes have been developed, such as the Integrated Microbial Genome system (IMG) [17], which has developed a system that allows clickable navigation of one or more genomes [18]. While the tool offers several alternative homology-based clustering methods, it does not have much flexibility in other aspects - for example, genes may only be organized into groups called “chromosomal cassettes” according to a hard-coded 300-bp intergenic distance threshold, and there is no way to export graphical representations of genome contexts.

Several tools have focused on visualization of syntenic and collinear regions, such as the plant genome duplication database [12], PLAZA [19], and Genomicus [20]. These tools are most appropriate when investigating plant and animal species, however, and could benefit from additional user flexibility and control in their visualization and interrogation of genomic segments. A number of genome navigable interfaces have been developed (such as the UCSC genome browser [21], the Gaggle genome browser [22], and JBrowse [23]). Many genome browsers have been developed with a focus of interrogating one or a few model organism(s) of interest, such as EcoCyc, (interrogating *Escherichia coli*[24]) and the Yeast Gene Order Browser (interrogating various species of yeast [25]). While these tools are sophisticated in their visualization schemes, they are limited in the species available for cross-species comparisons. MicrobesOnline [26] has developed a “domain browser” tool, which allows one to analyze the domain content of homologous proteins across microbial species. However, this tool compares the domain content of one gene at a time (rather than the organization of groups of genes) and so is not appropriate for studying changes in genomic context.

A tool with broad applicability, powerful multi-genome visualization tools, and a high degree of user control could complement the existing set of synteny and genomic context comparison tools well. To bridge the gap in genomic context comparison and visualization software, we have developed a new tool: JContextExplorer. Our tool extends the Java Multidendrograms package [27], which allows for flexible computation, re-analysis, and export of multidendrograms. We apply the multidendrogram approach to a set of user-supplied annotated genomes to create “context trees”: genomic contexts (which form the leaves of the tree) are assembled into a multidendrogram using variable group agglomerative hierarchical clustering. Previous genomic context investigations often determined the genomic contexts of interest in a set of species, and compared the observable differences in genomic contexts to a phylogenetic tree of the organisms [28-30]. However, genomic contexts do not always differ in ways that match species phylogeny, especially when a number of horizontal gene transfer events have taken place [30]. Our context tree approach offers an alternative to whole-species or even single gene phylogenetic trees that emphasizes the arrangement, size, and spacing of individual genetic elements within a contextual region of DNA instead of nucleotide-specific differences in the DNA.

The genomic contexts used to assemble context trees may be interrogated in an intuitive context viewer window, and information associated with individual genes may be retrieved by button clicks. Our software facilitates easy modification of parameters, and enables interrogation of several alternative genomic contexts of interest simultaneously. A balance of automation and manual control is essential for any software tool; we have attempted to automate only essential processes (such as tree computation and tree rendering), and leave a great deal of control to the user. Our motivation was to develop a novel, general-purpose genomic context comparison platform to both (1) generate context trees, and (2) facilitate genomic exploration through our multi-genome browser interface. We demonstrate a use case for our tool by resolving annotation ambiguities between ggt and hpxW genes among 22 species of alpha and gamma proteobacteria. Though in the use case provided here we focus on microbial species, we emphasize that analyses are not limited to microbial species.

## Implementation

JContextExplorer is a platform-independent pure Java application, requiring Java 1.6 or higher. The software extends the MultiDendrograms software package [27], and also uses BioJava [31] and the Java EPS Graphics2D API (version 0.1) [32]. The software has been tested to functionally equally on MacOS X, Windows 7, and Linux Ubuntu environments. Input data is read in via a series of tab-delimited text files. We provide instructions and examples in the user manual (Additional file 1) to help familiarize new users to the tool. The look and feel of all GUI components has been set to match the default look and feel of the operating system running the program. Program development was undertaken over several platforms to ensure an intuitive look and feel on all major platforms.

JContextExplorer has the ability to output JPG, PNG, and EPS representations of context tress and multi-genome browsable contexts. EPS representations of genomic contexts were achieved using the Java EPS Graphics2D API [32]. It took approximately 35 seconds to launch the program with a set of 22 annotated microbial genomes, computationally predict operons in all organisms using an intergenic distance threshold of 20 nucleotides, and load pre-computed homology cluster information for 81,102 annotated genes on a 2 x 2.8 GHz Quad-Core Intel Xeon processor, with 16 GB of RAM and total memory of 2 TB.

## Results

### JContextExplorer software usage

**Figure 1:**
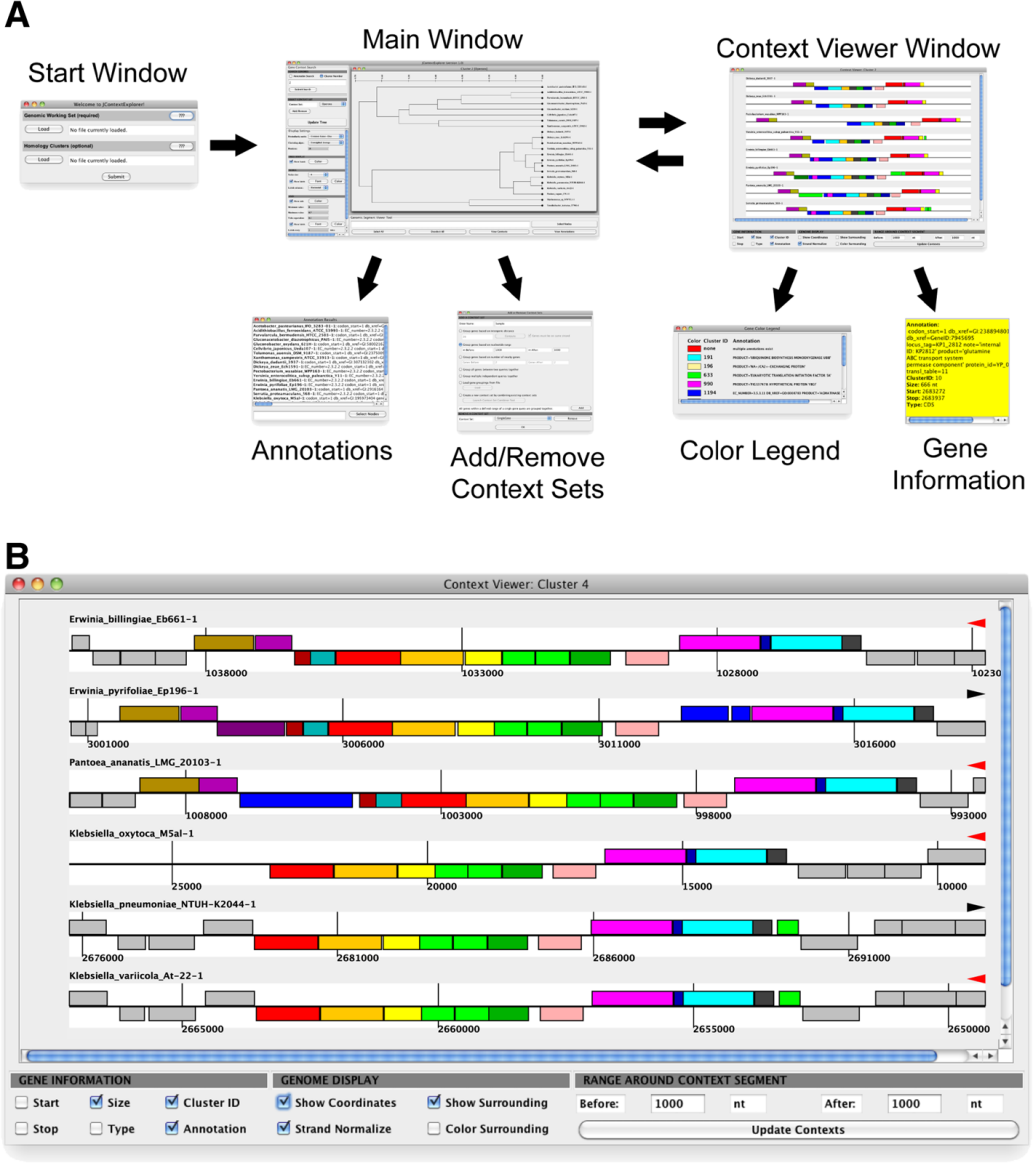
**Layout of JContextExplorer windows.** (**A**) JContextExplorer operates via usage of an initial data-loading frame, followed by coordination of a main window and multi-genome browser context viewer window (top). These frames have several associated child windows (second row): from the main window, a scrollable list of gene annotations (Annotations) and a window to facilitate multiple loading and switching between alternative context sets (Add/Remove Context Sets); from the context viewer window, an alphabetized gene color legend (Color Legend), and pop-up window of information relating to a specific gene (Gene Information). An enlarged view of the context viewer window (**B**) reveals a scrollable viewing area, where genes are rendered as colors rectangles appearing above or below a centerline, depending on their strandedness. Genes are colored according to homology or associated annotation. The Gene Information panel (lower left-hand corner) describes information to be delivered upon click (Gene Information window). Toggling checkboxes in the Genome Display panel (lower center) modifies the display of all rendered genomic segments. The range of the displayed region may be easily changed in the Range Around Context Segment panel (lower right-hand corner).

**Figure 2:**
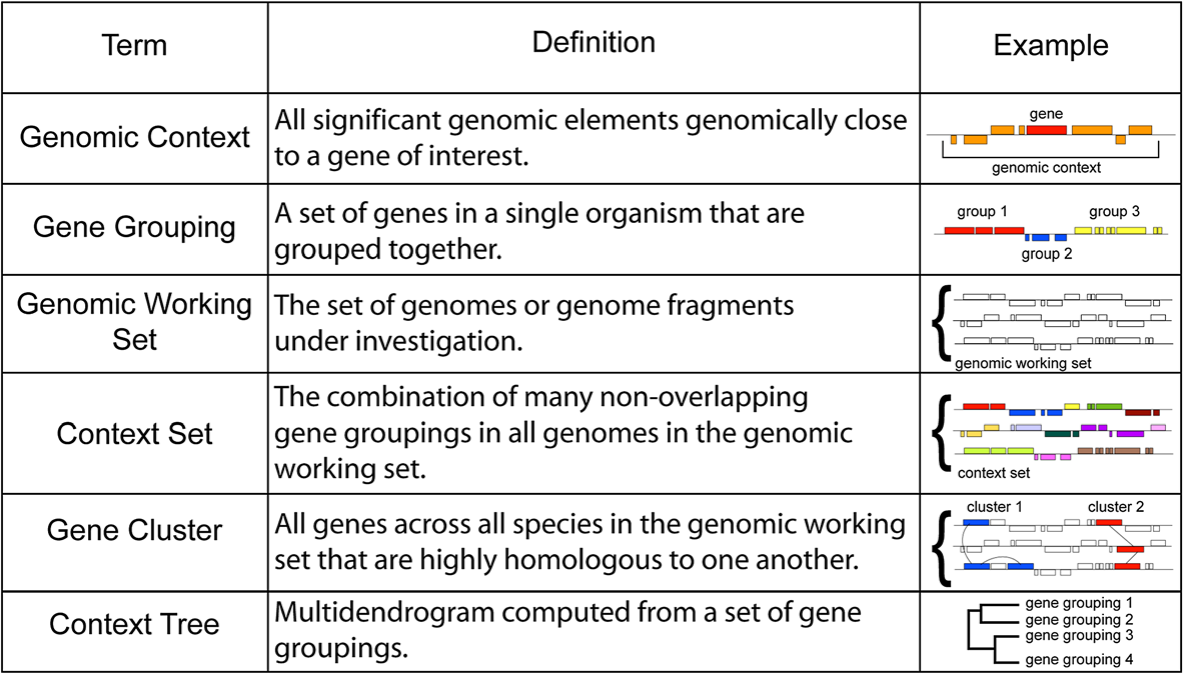
**JContextExplorer technical terminology.** Technical terms relevant to the JContextExplorer program (column 1) are defined (column 2) and demonstrated via graphical representation (column 3).

JContextExplorer may be launched via downloadable executable JAR file, or directly through the Internet via Java WebStart at http://www.bme.ucdavis.edu/facciotti/resources_data/software/. The program is organized as a series of major and minor windows laid out in a semi-hierarchical manner (Figure 1)**.** An initial welcome window invites the user to (1) specify the genomic working set (the set of genomes to investigate, see Figure 2) and (2) include cross-species homologous gene cluster information. Individual annotated genomes should be formatted as tab-delimited .GFF files (version 2). This information is imported into JContextExplorer by selecting either a directory containing a set of .GFF files or an additional tab-delimited mapping file listing the system locations of all individual annotated genomes files and corresponding species names. The user may also include tab-delimited cross-species gene clustering information, which could be computed using a combination of BLAST [33] and MCL [34], for example, or one of a number of various other gene clustering pipelines [35,36]. Homology cluster information may be entered in 5 alternative tab-delimited file formats (please see the user manual for a more detailed description). Once these files have been loaded, the user pushes a “submit” button to close the starting window and open the main window.

Once in the main window of the system, the user may search all loaded genomes by (1) gene annotation or (2) common homology group ID number. All computed genomic groupings in all organisms that contain one or more genes that match the search query are retrieved and organized in to a multidendrogram, according to a dissimilarity measure and linkage function. As a default, the starting context set defines genomic groupings only as the annotated features that match a search query (called the “SingleGene” context set), however 6 additional context sets are available, and may be accessed by clicking the “Add/Remove” button in the starting frame. Available genomic grouping schemes include organizing genes into operons, taking a range of nucleotides or genes around a query match, and loading a customized set of genomic groupings from file (for a complete description, please see the user manual). In this program, we have implemented 4 genomic grouping comparison metrics (or dissimilarity measures), each of which are appropriate for different use cases. If the genomic groupings that comprise a given context set are large, we suggest using either “Common Genes – Dice” or “Common Genes – Jaccard” metrics, which implement the set-based Dice and Jaccard dissimilarity approaches [37], with the individual annotated features within each grouping acting as elements and the whole genomic grouping acting as the set. If genomic groupings contain the same annotated features, however vary in the intergenic spacing between features, we recommend using the “Moving Distances” approach, which uses gene order and intergenic spacing to describe differences between contexts. Changes in intergenic spacing between genes within an operon has been experimentally shown to be related to gene co-expression in *E. coli* and *B. subilits*) [4], and may be a reflection of microbial gene regulatory networks changing over evolutionary time [3,4]. Finally, if the context set under investigation does not appear to change significantly except in the size of one or more genes, the “Total Length” dissimilarity metric may be effective (this is especially useful in for genomic groupings that consist of only one or a few genes). A more detailed description of these dissimilarity metrics is available in the user manual.

Linkage methods and display options available in the original multidendrograms package [27] are re-implemented here, which allows for easy re-computation of the context tree. All generated trees appear as individual internal frames; the user may therefore work on several alternative contexts at once (changes in tree computation and rendering will affect only the tree in focus). Individual leaves on the tree (which each represent a single context set grouping) are named by concatenating the name of the organism from which they derive to a serial number of the instance that a query match was found within that organism. Individual leaves on the tree may be selected by clicking on their name, clicking the “select all” button, or entering a leaf name search filter in the genomic context viewer tool search bar (located below the tree). Subsequent mouse clicks may bring up child windows either for (1) annotations of the query matches for selected, or (2) a multi-genome browser window (context viewer window). As depicted in (Figure 1A), the start window, main window, and context viewer window are the central components of the tool, and various child windows are available within the main window and context viewer windows.

The context viewer window **(**Figure 1B**)** is a multi-genome browser specifically designed to interrogate analogous gene groupings across many species (or multiple genomic regions within a single species), rather than explore the genome of a single species. Individual genes are rendered as colored rectangles, oriented above or below a centerline to represent their placement on the forward (above centerline) or reverse (below centerline) strand. Each segment is centered about the center of each gene grouping. Below all rendered contexts, a “genomic display” sub-panel contains check box options to (1) show/hide genomic coordinates, (2) normalize displayed contexts according to strand (which may allow for easier visual inspection of analogous contexts), (3) display genes surrounding each context that are not a part of the context, and (4) color the genes surrounding the context (if this is unchecked, surrounding genes are displayed as gray). Genes are colored according to homology or common annotation, depending on the method used to generate the context tree. Left clicking on individual genes within a rendered context brings up a pop-up window displaying biological information related to each gene (this information may be modified in a “gene information sub-panel”). Right clicking enables exporting rendered contexts as an image and offers the option to display a gene color legend. Middle clicking selects the clicked gene as well as all homologous genes or genes that with the same annotation (depending on the initial search type) displayed in the frame. Finally, the rendered range of each context may be easily changed using the “range around context segment” sub-panel, and clicking an “update contexts” button. The context viewer window and main window are actively linked; modifying selected leaves in the tree, for example, will add or remove these leaves in the context viewer window after clicking the “update contexts” button. The tool is designed to facilitate coordination of the context tree and the context viewer window – such coordination may inspire re-investigation of the same gene of interest using alternative context groupings, or re-computation of the context tree using a different clustering algorithm.

### Analysis of the hpxW and ggt genes in 22 alpha and gamma proteobacteria

In the gamma-proteobacterial species *Klebsiella oxytoca M5a1*, the *hpxW* gene is known to form an operon with *hpxW*, *hpxY*, and *hpxZ*[38]. The *hpxW* gene, however, is highly homologous to another gene encoding gamma-glutamyl transpeptidase (*ggt*). A sequence alignment of *K. oxytocahpxW* and *Escherichia coli* ggt revealed that their amino acid sequences are almost co-linear and share 30% identity. This high degree of homology confuses automated annotation programs, which often misannotate *hpxW* as *ggt*. Fortunately, the ggt enzyme has been characterized in several microbial organisms, [39] and has a genomic context very different from the hpxW context (*ggt* occurs as a single gene, *hpxW* in an operon with at least 3 other genes). Therefore, by taking into account context as well as homology, it is possible to accurately separate *ggt* genes from *hpxW* genes.

**Figure 3:**
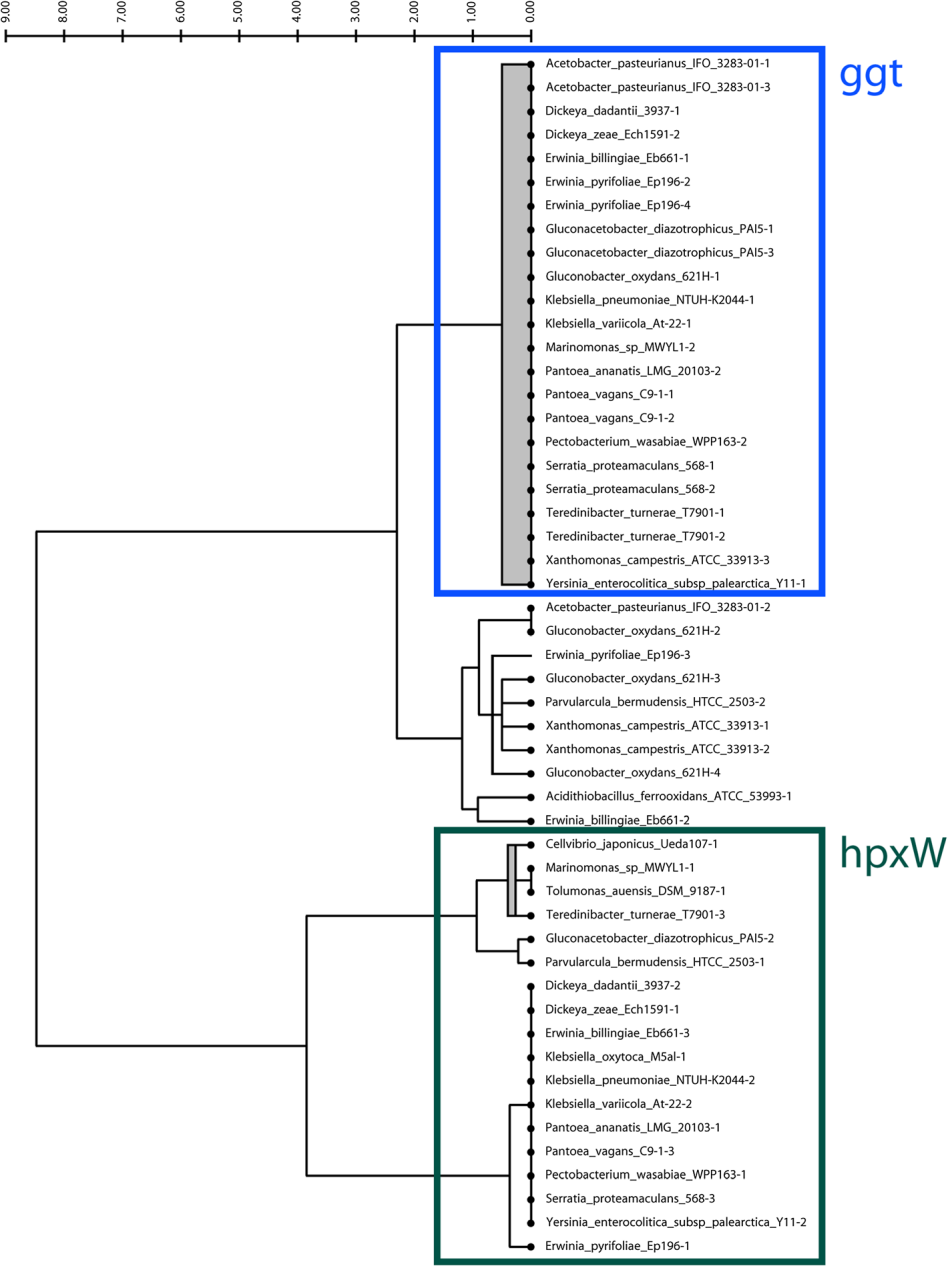
***hpxW *and *ggt *context tree.** Clusters of all homologous gene clusters in 22 alpha and gamma proteobacterial species were constructed using BLAST [33] and tribe-MCL [34]. All *ggt* and *hpxW* genes naturally grouped into the same homology cluster. Using JContextExplorer, we defined a context set, which we named “D75”, that placed all genes on the same strand within 75 nucleotides of each other into common gene groupings. We constructed a context tree of the ggt/hpxW homology cluster using the “Common Genes – Dice” dissimilarity metric and “Joint Between-Within” linkage function (above). The data segmented into two branches, one of which corresponded to the previously described hpxW context (green box), and the other into a combination of the ggt context (blue box) and an undetermined third group. Manual inspection of individual contexts in the third group might reveal that some members of this group belong with the ggt group, and some with the hpxW group. Additionally, some members in this third unknown group could represent “transitional” cases between the *hpxW* and *ggt* gene (a gene that performs the functions of both *hpxW* and *ggt*, for example). JContextExplorer’s context viewer tool proved helpful in manually interrogating this third group.

We used JContextExplorer to attempt to separate *ggt* genes from *hpxW* genes in 22 alpha and gamma proteobacterial species based on differences between *ggt* and *hpxW* contexts. We found that ggt and hpxW grouped into two major out-branches (Figure 3). Interestingly, we discovered a third group, where manual investigation revealed that it was unclear if these genes were *ggt* or *hpxW* (data not shown). Visualization of the contexts in the hpxW group revealed agreement with previously described *hpxWXYZ* structures, and a comparison of a whole-genome phylogenetic tree with the ggt / hpxW context tree (Additional file 2) revealed good agreement among closely related organisms. Details relating to the methods associated with the above analyses are also available (Additional file 3). This investigation highlights the utility of combining automation (generating the ggt/hpxW context tree) with manual interrogation (investigation using the multi-genome browser context viewer tool).

## Conclusion

Comparing genomic contexts across organisms is an effective but underutilized technique. While a handful of custom approaches have been developed, no universal platform for cross-species genomic context analyses has yet been produced. We have developed JContextExplorer to address this need. We have attempted to make JContextExplorer easy to install and use by offering our program as a GUI WebStart application (launching is as simple as navigating to a website, and clicking on the appropriate button). Additionally, our program is organized in a way that does not require a steep learning curve among prospective users. To help new users, we provide an extensive user manual and a series of video tutorials (Additional file 1) along with the program executable (Additional file 4). We hope that JContextExplorer may find use in the bioinformatics community with its emphases of producing a positive user experience and simultaneously offering a navigable tool of high quality and portability.

## Availability and requirements

**Project Name:** JContextExplorer

**Operating System:** Platform independent

**Programming language:** Java

**Other requirements:** None.

**License:** Source code and binary executable are available under terms of the GPL free software license (ver-sion 2 or later) at http://www.bme.ucdavis.edu/facciotti/resources_data/software/. Incorporation into commercial software under non-GPL terms is possible by obtaining a custom license from the University of California.

**URL:**  http://www.bme.ucdavis.edu/facciotti/resources_data/software/.

## Competing interests

The authors declare they have no competing interests.

## Authors’ contributions

PS wrote the source code and drafted the manuscript. PS and TH analyzed the hpxWXYZ operon in alpha and gamma proteobasterial species with TH, which was instrumental in the development of JContextExplorer. TH also helped write the background information regarding purine catabolism and the hpxW gene in alpha and gamma proteobacteria, in the supplemental information. MF provided essential feedback for software development and oversaw the project, and helped to interpret results related to the hpxWXYZ genomic contexts. All authors contributed to the preparation of the manuscript, and have read and approved the final manuscript.

## Supplementary Material

Additional file 1 ContextExplorer User Manual. Comprehensive user manual, including installation / use instructions, examples, diagrams, contact information, and links to website, tutorial videos, source code, and other resources. Click here for file

Additional file 2 **Supplementary Figures and Methods related to separating hpxW from ggt in 22 alpha and gamma proteobacteria.** Visualization of hpxW contexts, comparison of whole-species phylogeny to hpxW sub-portion of JContextExplorer-generated context tree, and detailed description of methods associated with hpxW/ggt analysis. Click here for file

Additional file 3 **Alpha and Gamma proteobacteria biological information.** Annotated genomes of 22 alpha and gamma proteobacterial species, whole-species phylogenetic tree for 22 alpha and gamma proteobacterial species, and MCL-tribe determined homology clusters for 22 alpha and gamma proteobacterial species. Click here for file

Additional file 4 **JContextExplorer version 1.07. Executable JAR file of the latest version of the JContextExplorer program.** Program may be launched on any computer with the Java runtime environment (JRE) installed. The program may be launched either from a command line or by double-clicking on the program icon. Click here for file
