# Supplementary material for: JContextExplorer: a tree-based approach to facilitate cross-species genomic context comparison
Source: BMC Bioinformatics. 2013 Jan 16;14:18. doi: 10.1186/1471-2105-14-18 (PMC3560190; doi:10.1186/1471-2105-14-18)
Supplement: Additional file 1 — ContextExplorer User Manual. Comprehensive user manual, including installation / use instructions, examples, diagrams, contact information, and links to website, tutorial videos, source code, and other resources. [file 1471-2105-14-18-S1.pdf]

JContextExplorer User Manual

Phillip Seitzer

Facciotti Lab
UC Davis

November, 2012
Version 1.07

TABLE OF CONTENTS

CHAPTER 1: GETTING STARTED 3

What is JContextExplorer? 3

Why should I use JContextExplorer? 4

CHAPTER 2: LAUNCHING JCONTEXTEXPLORER 6

Where I can find JContextExplorer? 7

What do I need to do before I can launch JContextExplorer? 7

CHAPTER 3: USING JCONTEXTEXPLORER 8

Summary of available features 9

Start Window 10

Genomic Working Set Input Instructions 11

GFF File Type Import Settings 13

Homology Cluster Input Instructions 15

Main Frame 18

Searching a Genomic Working Set 20

Editing Tree Display and Construction Settings 21

Available Pairwise Dissimilarity Metrics 21

Launching Subordinate Windows and Selecting Nodes 25

Add/Remove Context Sets 26

Available Context Set / Genomic Grouping Types 27

View Annotations 30

Context Tree Frame 31

Left Click Options 33

Right Click Options 34

Multi-Genome browser / Context Viewer 36

Gene Information sub-pane 37

Genome Display sub-pane 37

Range to Display sub-pane 38

Left-click options 39

Right-click options 40

Middle-click options 40

Gene Color Legend 41

CHAPTER 4: ADDITIONAL RESOURCES 42

Tutorial I: Determining HpxW from GGT in 22 alpha and gamma-proteobacteria 43

Tutorial 2: Comparing a JContextExplorer-generated context tree to a phylogenetic tree 47

Video tutorials 49

Author Contact Information 51

CHAPTER 1: GETTING STARTED

WHAT IS JCONTEXTEXPLORER?

JContextExplorer is a tool to facilitate cross-species genomic context comparisons, based on previously determined annotated genomes (and possibly homology clusters). JContextExplorer uses variable-group agglomerative hierarchical clustering to create "context trees", where each leaf represents a single gene neighborhood.

JContextExplorer offers several ways to (1) define genomic groupings (i.e., create the objects to be clustered), (2) perform pairwise comparisons (compare each object to be clustered with each other object to be clustered, and (3) assemble these comparisons into a tree (link individual dissimilarities using standard clustering approaches). JContextExplorer also allows for fast searching a set of annotated genomes, as well as several flexible visualization tools.

As evident in the name, JContextExplorer is designed to facilitate exploration. Each of the three major steps in context tree creation (genomic grouping definition, pairwise comparisons, tree creation) may be re-computed quickly and easily with alternative parameters. The graphical interface is designed for point-and-click investigation, and provides fast and easy export of major results (context trees, multi-genome context renderings, etc). We strongly suggest using the automated features (tree computation) in concert with the manual interrogation features (multi-genome browser) in your investigations.

WHY SHOULD I USE JCONTEXTEXPLORER?

There are many reasons to use JContextExplorer. These reasons tend to fall into two categories: (1) Reasons relating to genomic context (or gene neighborhood) comparison and (2) reasons relating to annotated feature exploration. Within the umbrella of genomic context comparison, one might be interested in

(1) Resolving ambiguities in annotated features.

(2) Comparing changes in gene regulatory network structure (as in the case of operons in microbial species).

(3) Discovering and interpreting potential horizontal gene transfer events.

(4) Within a set of duplicated homologous genes across species, determining which copies are ancestral and which represent more recent expansions.

Within the umbrella of genome exploration, JContextExplorer's GUI interface allows one to

(1) Peruse annotated features nearby to a gene or genes of interest.

(2) Compare (and count) textual annotations within a set of homology clusters.

(3) Effectively merge one or more context sets into superclusters.

These are but a short list of suggested uses. Any comparative genomic analysis that could benefit by alternative methods of organization and visualization of multiple genomes (or section of multiple genomes) stands to benefit from JContextExplorer. For a few examples of some biologically interesting uses, please see Chapter 4: Additional resources.

CHAPTER 2: LAUNCHING JCONTEXTEXPLORER

Where I can find JContextExplorer?

JContextExplorer can be found on the software Facciotti lab website:

http://www.bme.ucdavis.edu/facciotti/resources_data/software/

On this website, JContextExplorer is available both (1) as a Java WebStart and (2) as a downloadable zipped directory. Supplementary documentation, instructions, and links to video tutorials may also be found on this page.

JContextExplorer is distributed as an executable JAR. However, it is also possible to build the tool from source. All source code is available on GitHub:

https://github.com/PMSeitzer/JContextExplorer

What do I need to do before I can launch JContextExplorer?

JContextExplorer runs on the Java Virtual Machine (JVM) version 1.6 or higher. If you do not have the Java runtime environment installed, please install the latest version of Java before attempting to launch JContextExplorer.

The Java Webstart version runs with a maximum heap size of 512 MB. Please make sure your system can accommodate for this memory allocation (if not, please use the zipped directory version instead of the WebStart version). If you are using the WebStart version, to launch JContextExplorer, simply click the WebStart launch button.

If you have downloaded a JContextExplorer in the zipped directory, you may either (A) double-click on the icon or (B) launch JContextExplorer from the command line with the following command:

```
java -jar <path-to-file>/JContextExplorer.jar
```

You may want to launch java with a larger max heap size to avoid memory-related problems. In that case, type the following command:

```
java -Xmx256M -jar <path-to-file>/JContextExplorer.jar
```

or
```
java -Xmx512M -jar <path-to-file>/JContextExplorer.jar
```

CHAPTER 3:

USING JCONTEXTEXPLORER

SUMMARY OF AVAILABLE FEATURES

JContextExplorer is organized as a series of major and minor windows laid out in a semi-hierarchical manner:

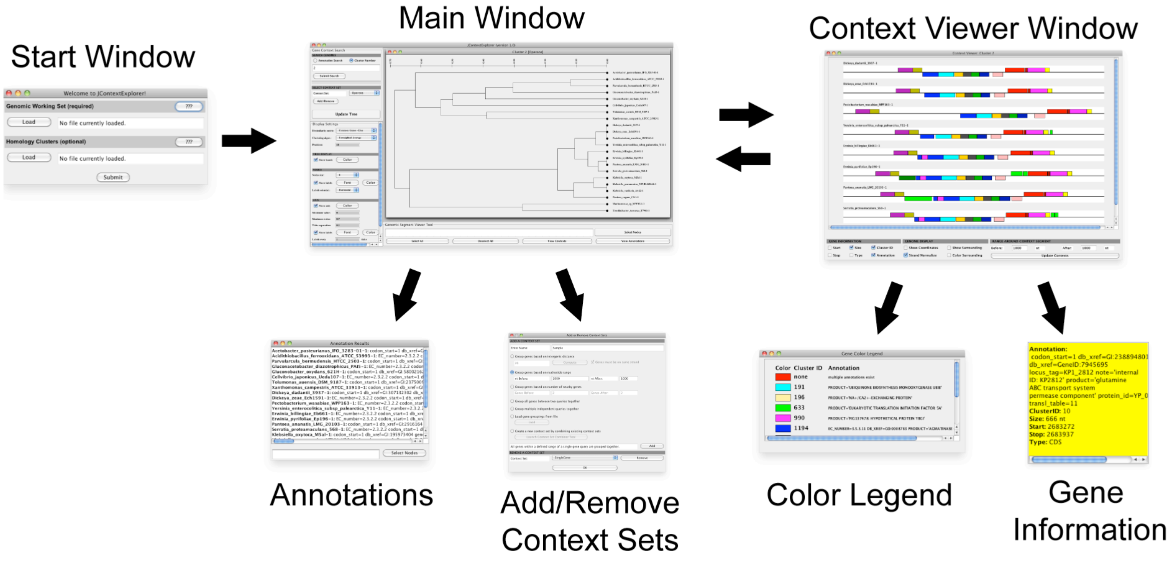

From an initial starting frame, a main window is launched. This main window has several child windows, including the Context Viewer (or multi-genome browser) window, which also has several child windows. Please see the instructions associated with each window for more information.

START WINDOW

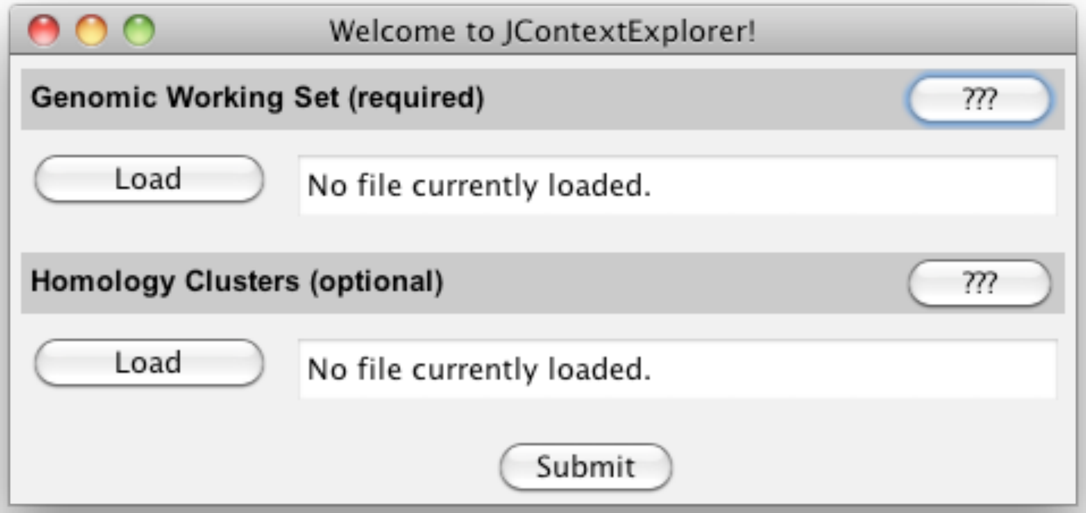

This is the starting window. All input files should be entered at this point. Input files consist of **(1) The Genomic Working Set files/directory**, **and (2) Homology Cluster files**. In each case, upon clicking the "Load" button, a file chooser will appear, allowing the user to select the appropriate file / directory. Once appropriate the files/directory have been loaded, please push the "submit" button to proceed to the main frame.

Genomic Working Set Input Instructions:

A Genomic Working Set is a collection of annotated genomes. When performing searches in JContextExplorer, JContextExplorer will query all genomes in the loaded genomic working set.

To load a genomic working set, push the "load" button below and either (1) Select a directory containing individual annotated genome files or (2) Select a genomic working set file.

Individual annotated genomes should be formatted in General Feature Format (or .GFF) [version 2], a standard tab-delimited text file format. GFF files should have the file extension ".gff".

Each line in the GFF file describes a single annotated feature, and is split into 9 columns. This program only reads in columns 1, 3, 4, 5, 7, and 9, which contain the following information:

Column 1: Sequence name

Column 3: Feature Type

Column 4: Feature Start Position

Column 5: Feature End Position

Column 7: Strand

Column 9: Annotation

If you specify a directory of .GFF files, JContextExplorer will name each genome according to the name of the file.

For example,

/SomeDirectory/CollectionOfGenomes/Organism1.gff will be named **Organism1.**

Please avoid names containing white spaces (instead, use underscores).

Instead of specifying a directory of .GFF files, you may specify a single genomic working set file. This file must be a 1 or 2-column tab-delimited text file. In the first column, please specify the file path to all annotated genome files you would like to include in your genomic working set. If you do not include a second column, each genome will be named according to the name of the file. The optional second column consists of a customized name for each genome.

WARNING!

When specifying file paths of individual genome files, please be sure to either specify (1) The absolute path or (2) The path relative to the directory from which JContextExplorer was launched. JContextExplorer will be unable to import files if the file paths are not correctly specified.

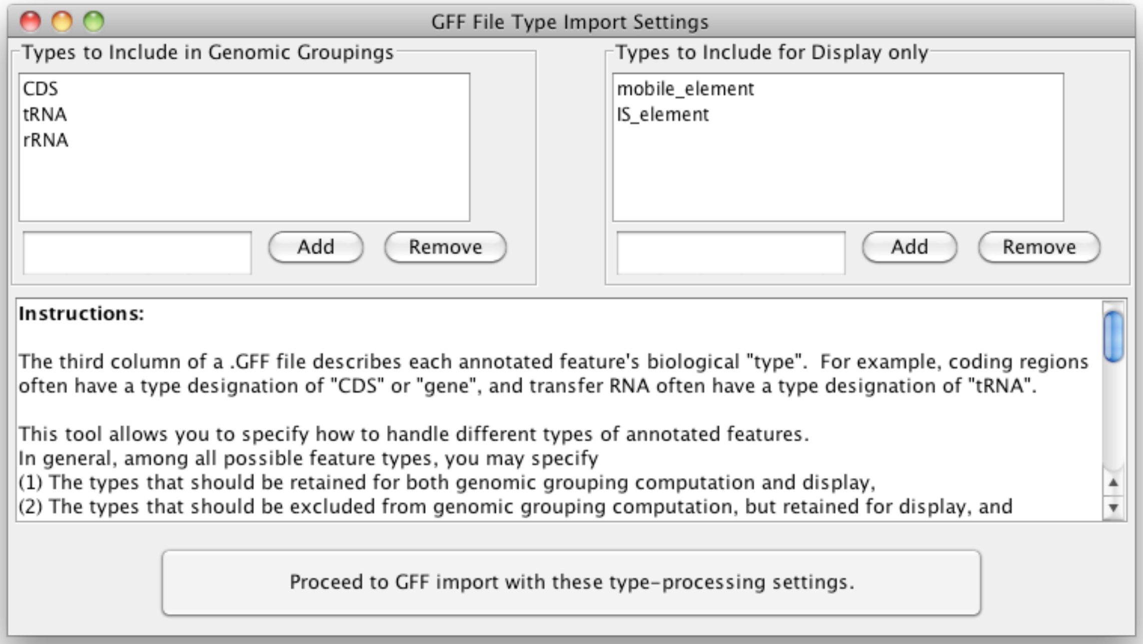

GFF File Type Import Settings

The third column of a .GFF file describes each annotated feature's biological "type". For example, coding regions often have a type designation of "CDS" or "gene", and transfer RNA often have a type designation of "tRNA".

This tool allows you to specify how to handle different types of annotated features.

In general, among all possible feature types, you may specify

(1) The types that should be retained for both genomic grouping computation and display,

(2) The types that should be excluded from genomic grouping computation, but retained for display, and

(3) The types that should be excluded altogether.

Types in the list **Types to Include in Genomic Groupings** (left) will be retained for both genomic grouping computation and display. Types in the list **Types to Include for Display only** (right) will be retained for display only when viewing genomic segments. **All other types will be ignored (excluded altogether).**

To add types to a list, type in the type in the text field below the list and push the "Add" button.

To remove types from a list, select the type with your mouse, and push the "Remove" button.

To transfer types from one list to another, select the type with your mouse, and drag the type to the other list.

WARNING!

Features in the GFF file may not overlap in the genomic coordinates they span. In the case that they do overlap, JContextExplorer will exhibit unpredictable behavior and likely fail.

Please ensure that no annotated features overlap prior to loading GFF files.

Homology Clusters Input Instructions:

Within a single genomic working set, certain annotated features may be homologous to one another. This may occur both within a single species and across multiple species. A group of homologous features is often referred to as a Homologous Gene Cluster, or simply a Homology Cluster. Numerous methods exist to detect homology across and within genomes, and to cluster annotated features in a set of genomes into homology cluster groups. Often, but not necessarily, these homology cluster groups are non-overlapping. That is, each annotated feature may belong to a maximum of one homology cluster.

For all homology cluster-associated processes, JContextExplorer assumes non-overlapping homology clusters.

When JContextExplorer searches for annotated features in a genomic working set, it may do so either by (1) Matching a textual query to individual genomic feature annotations or (2) Matching a homology cluster ID number.

Textual annotations may be unreliable (especially if a genomic working set contains genomes annotated by different groups), so it may be worthwhile to compute homology clusters and load these computed homology clusters into JContextExplorer.

WARNING!

JContextExplorer cannot compute homology clusters from a set of sequenced genomes, only search a set of pre-computed, loaded homology clusters.

To load a set of pre-computed homology clusters, click the load button below the banner, and select the appropriate file. Homology clusters may be defined according to gene name or precise feature coordinates. All files must be tab-delimited, and each line in the file describes an individual feature - homology group relationship. Depending on the number of columns provided, each line is parsed differently. Lines in the file that do not follow the specifications described below will be ignored.

There are 5 acceptable line formats:

(1) Five-Column Format

If there are 5 tab-delimited entries in the line, entries take on the following values:

Column 1: Genome Name

Column 2: Sequence Name

Column 3: Feature Start Position

Column 4: Feature End Position

Column 5: Homology Cluster ID Number

If a feature starts at Feature Start Position and stops at Feature Stop Position, on the sequence named Sequence Name, in the genome named Genome Name, this feature is assigned the provided Homology Cluster ID Number.

(2) Four-Column Format

If there are 4 tab-delimited entries in the line, entries take on the following values:

Column 1: Genome Name

Column 2: Sequence Name

Column 3: Annotation Key

Column 4: Homology Cluster ID Number

If a feature contains the string Annotation Key in it's annotation, and is found on the sequence named Sequence Name in the genome named Genome Name, this feature is assigned the provided Homology Cluster ID Number.

In the Annotation Key field, please use underscores instead of spaces.

(3) Three-Column Format

If there are 3 tab-delimited entries in the line, entries take on the following values:

Column 1: Genome Name

Column 2: Annotation Key

Column 3: Homology Cluster ID Number

This format is identical to Four-column format, however does not check for agreement in the sequence name.

(4) Two-Column Format

If there are 2 tab-delimited entries in the line, entries take on the following values:

Column 1: Annotation Key

Column 3: Homology Cluster ID Number

All features in all genomes in the genomic working set with an annotation that contains the Annotation Key are assigned the provided Homology Cluster ID Number.

(5) Single Column Format

If there is only a single entry in the line, this entry is taken to be the Annotation Key. All annotated features that contain the annotation key are given a homology cluster ID number, which is determined by the line number in the file.

MAIN FRAME

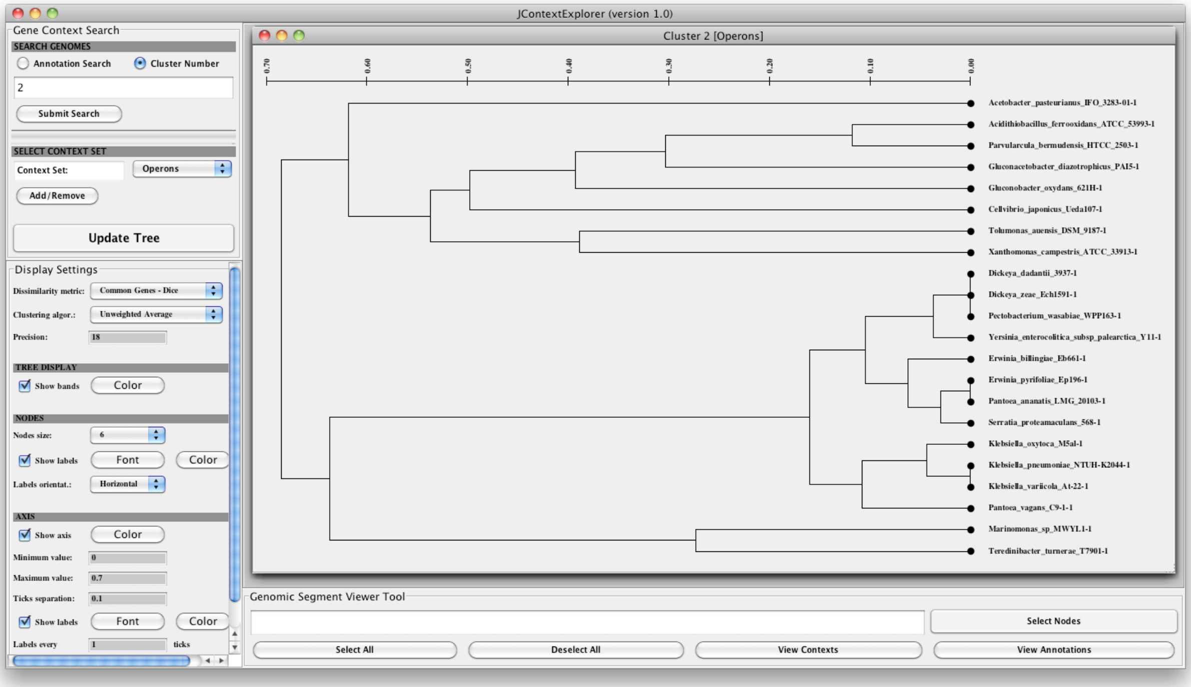

This is the main window. From this window, you may (1) build context trees by querying your genomic working set and build context trees (2) edit tree display and construction settings (3) select one or more nodes in a currently active context tree frame or (4) Launch the view annotations and/or multi-genome browser/context viewer window(s).

Searching a Genomic Working Set

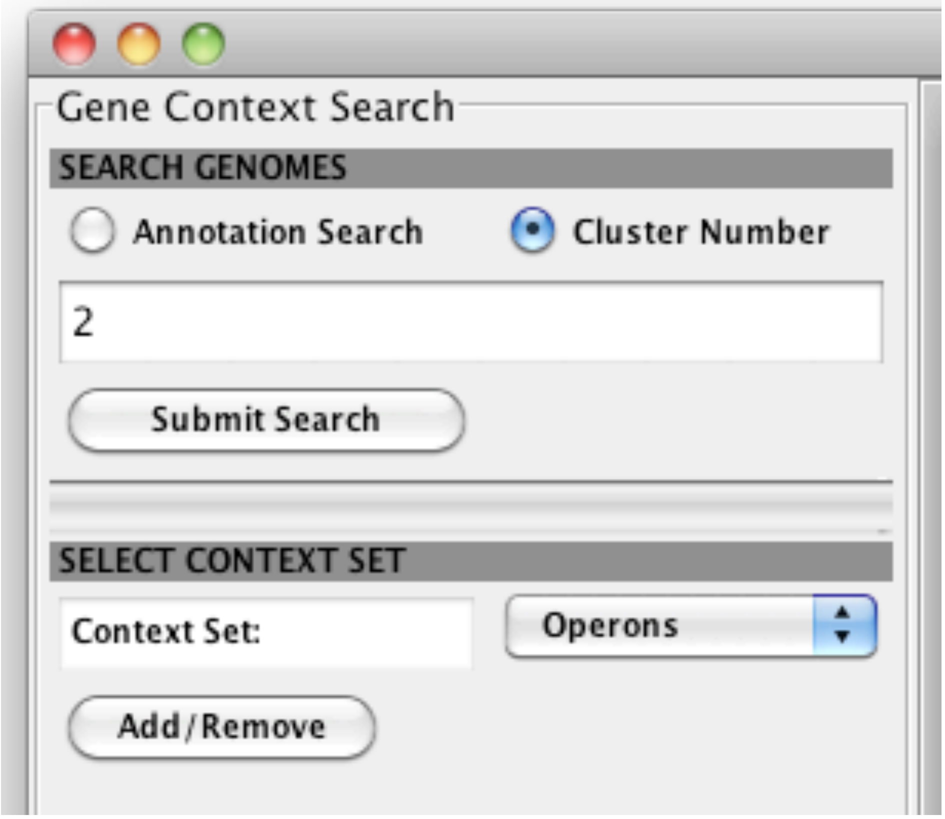

Type your search in the search bar (shown above). If you select the **Annotation Search** radio button, JContextExplorer will search through the associated feature annotation for **exact, case-insensitive matches**. All genomic groupings that contain one or more annotated features that match the textual query are retrieved, compared, and assembled into a context tree. If instead you select the **Cluster Number** radio button, JContextExplorer will search through associated loaded homology cluster ID numbers for an **exact match**.

If you would like to specify multiple queries (the equivalent of an "OR" statement), **separate your search queries using a semicolon**. For example, a search of "3" with the cluster number radio button selected will search for all annotated features with a cluster ID number of 3, a search of "3; 51" will return all annotated features with a cluster ID number of 3 **OR** 51.

Under the banner **Select Context Set**, there is a drop-down menu allowing you to choose which context set you would like to use for your search. **A Context Set is**

a set of genomic groupings. A genomic grouping is simply a grouping of genes that exist on the same genome. These groupings are the elements that form the leaves of all generated context trees.

A context set describes how genomic neighborhoods should be defined. There are many ways to describe context sets. As a default, JContextExplorer will create a context set called “SingleGene”, which consists only of the annotated feature that matches the search query. You may add, remove, and manage context sets by clicking the “Add/Remove” button below the Select Context Set banner (shown above). A detailed description of available ways to define context sets is available in the “Add/Remove Context Sets” section **(page XXX)**.

Editing Tree Display and Construction Settings

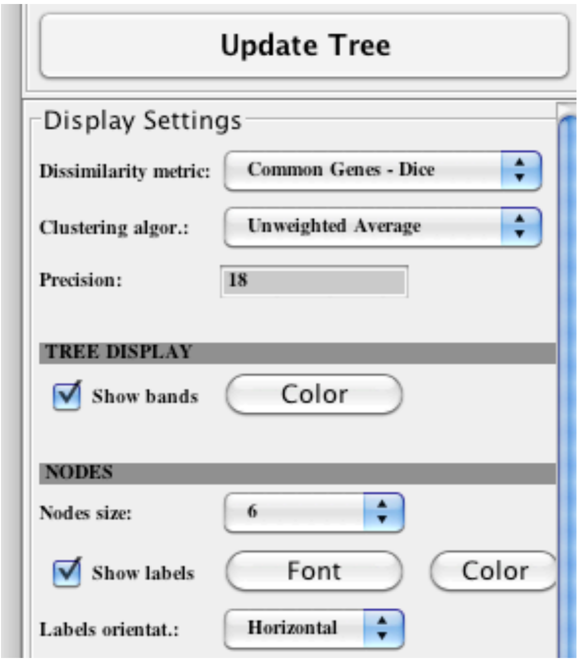

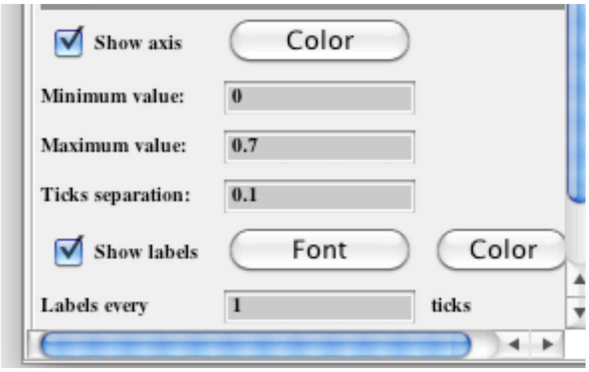

Context Trees may be edited in various ways, following their computation, both in terms of (1) their construction and (2) their display. The majority of features in this tool were previously developed in the Multidendrograms software package:

Gomez, S., Fernandez, A., Montiel, J., & Torres, D. (n.d.). Solving Non-Uniqueness in Agglomerative Hierarchical Clustering Using Multidendrograms. *Journal of Classification*, *65*, 43-65. doi:10.1007/s00357-008-

A complete user manual is available at http://deim.urv.cat/~sgomez/multidendrograms.php#description

Please refer to this documentation for more information.

Available Pairwise Dissimilarity Metrics

A feature that exists in this tool that did not exist in the previous multidendrograms package is the various ways to compute pairwise dissimilarities between genomic groupings.

(1) Common Genes – Dice

All common genes are identified between two genomic groupings. Common genes are defined either by common cluster ID number (if the

search carried out is homology cluster - based) or annotation (if the search carried out is annotation – based). The pairwise dissimilarity between gene groupings X and Y is computed according to the Dice Formula:

d = 1 – ((2 * |X AND Y|) / (|X| + |Y|))

(2) Common Genes – J'accard

All common genes are identified between two genomic groupings. Common genes are defined either by common cluster ID number (if the search carried out is homology cluster - based) or annotation (if the search carried out is annotation – based). The pairwise dissimilarity between gene groupings X and Y is computed according to the J'accard Formula:

d = 1 – ((|X AND Y|) / |X OR Y|))

(3) Moving Distances

In microbial genomes, co-transcribed features are often grouped into same-stranded positionally adjacent groupings (operons), with little intergenic spacing between them. As the spacing between individual features widens, this could indicate a change in the transcriptional processing of a genomic grouping: for example, a large widening in the center of a tightly packed gene grouping could indicate the splitting of one operon into two. Also relevant to this comparison is a rearrangement of genes within a single operon: gene order in operons may convey information about the relative importance of transcribed products. This pairwise comparison metric attempts to capture these behaviors, through a weighted sum of observed differences (penalties) between two genomic groupings X and Y.

The Moving Distances approach is designed to compare genomic groupings that contain **the same set of homologous genes**. If there is even one inclusion/exclusion, the two groupings with score a dissimilarity value of **1** (maximum dissimilarity).

Provided that for every gene in gene grouping X there exists a homologous gene in gene grouping Y, inversions / gene rearrangements between the groupings are assessed. A single rearrangement incurs a dissimilarity penalty of **0.2.** If rearrangements have occurred, the rearrangements are counted, and a dissimilarity measure is returned. Therefore, if 5 or more rearrangements are counted, genomic groupings are returned with a dissimilarity score of 1 (maximum dissimilarity).

If no rearrangements have occurred, distance-widening-based penalties are then assessed.

If no widening has occurred between analogous genes across genomic groupings, no penalty is incurred.

If a **slight widening** (between 10 and 25 nt) has occurred, this incurs a dissimilarity penalty of **0.02**

If a **medium widening** has occurred (between 25 and 200 nt) has occurred, this incurs a dissimilarity penalty of **0.05**

If a **large widening** has occurred (greater than 200 nt) has occurred, this incurs a dissimilarity penalty of **0.2**. Note that this widening often signifies a gene insertion.

In future versions of this software, more user control will be allowed to modify the various penalties assessed, and the values to associated with these penalties. The set of penalties/penalty values included here are designed to be effective for **comparing highly phylogenetically similar organisms** (within the same phylum), or else **highly conservative genomic groupings.**

d = SUM(penalties)

(4) Total Length

The total size of each genomic grouping X and Y is computed by taking the distance from the start of the earliest annotated feature to the stop of the

latest annotated feature. The dissimilarity is taken to be the average difference:

d = (2 * ABS(|X|-|Y|) / (|X| + |Y|)

Launching Subordinate Windows and Selecting Nodes

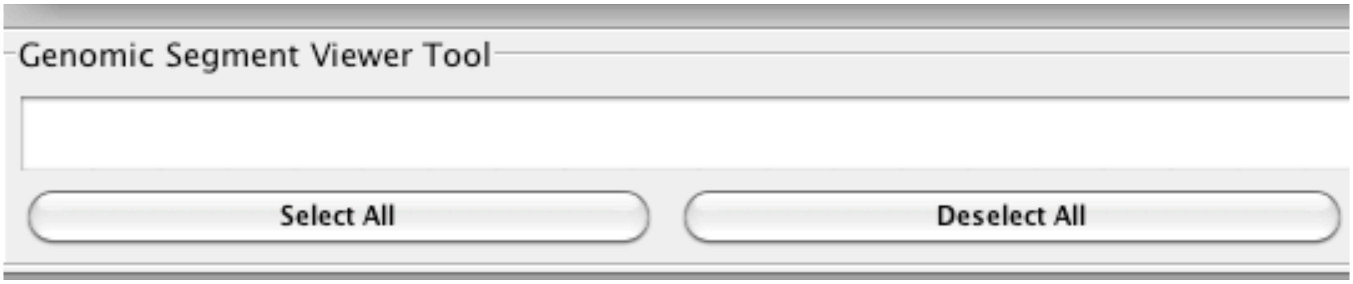

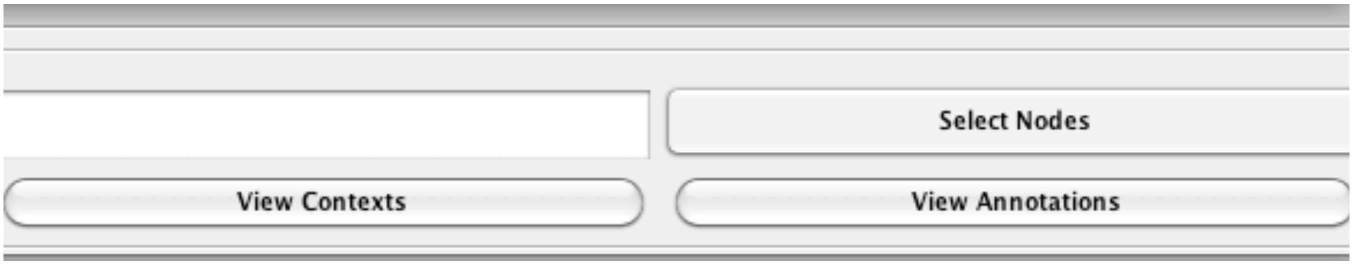

Typing into the text field directly under the “Genomic Segment Viewer Tool” banner allows for selection of appropriate leaves in the active context tree frame. All nodes that contain the search text will be selected. To select nodes, type your query, and push the **Select Nodes** button, or strike the enter key.

Please note that you may also select leaves by clicking directly on the leaf name, as explained in the “Context Tree Frame” section **(page XXX)**.

If you would like to specify multiple queries (the equivalent of an “OR” statement), **separate your search queries using a semicolon or white space**. For example, if you would like to select all nodes that contain the text “coli” or “subtilis”, type “coli subtilis” or “coli; subtilis”.

To launch the view annotations frame, push the **View Annotations** button, with the appropriate leaves selected. This will display the associated annotation of all query matches within the genomic grouping associated with that leaf. Similarly, you may launch the multi-genome browser by pushing the **View Contexts** button, with the appropriate leaves selected.

ADD/REMOVE CONTEXT SETS

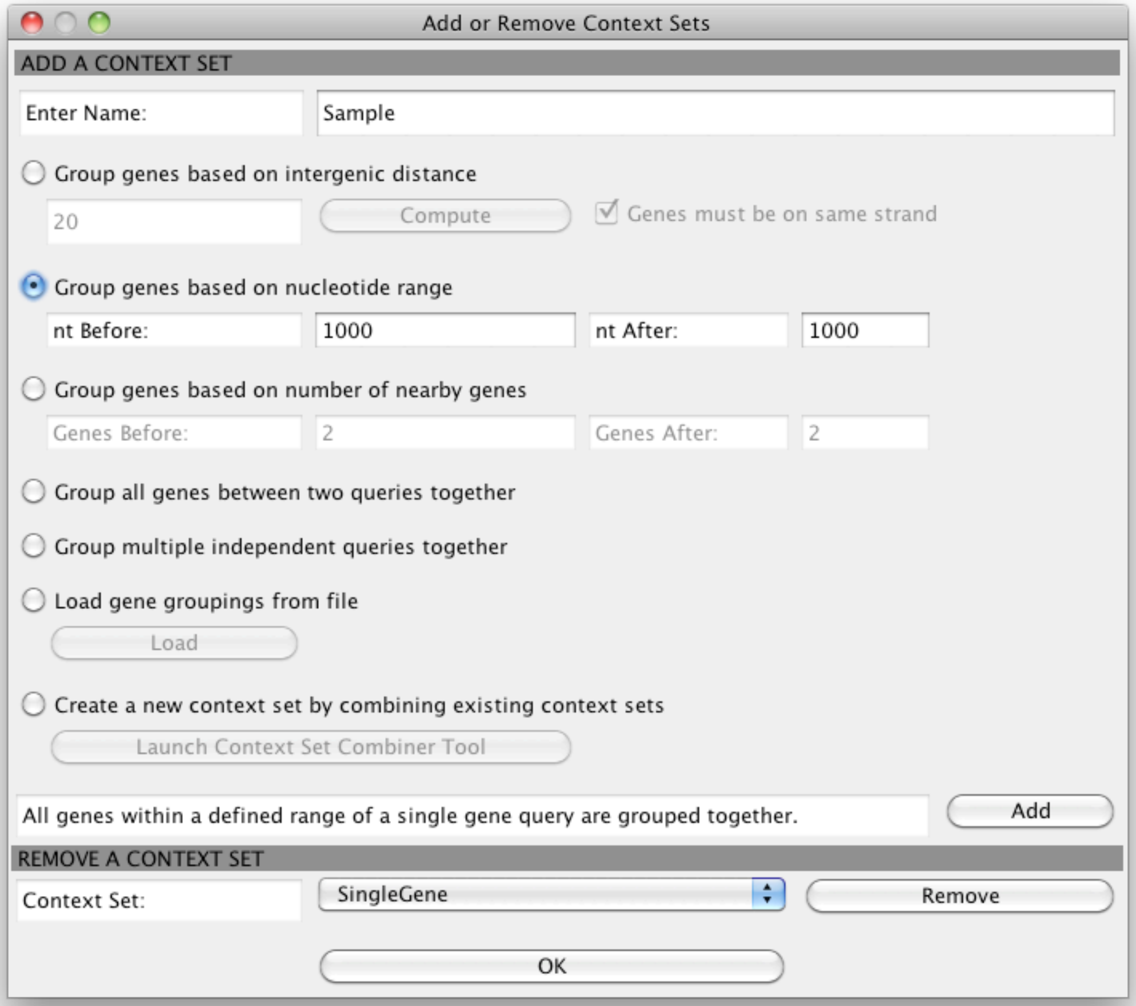

This is the add/remove context sets frame. From this frame, you may define new genomic grouping protocols, or remove unwanted existing genomic grouping protocols. To add a new genomic grouping protocol, first type a name for your new genomic grouping in the **Enter Name: text field**. Then, select the appropriate scheme for genomic grouping computation (all available schemes described below). Once your set is ready to add, you will see a message appear in the text field next to the "add" button. Click the "add" button to add this to the set of existing genomic grouping methods. This new method will appear in the drop-down list of existing context set definitions under the **Remove a context set**

banner. You may remove sets by selecting them from this same drop-down list, and clicking the **Remove** button. Once you have finished adding/removing/managing context sets, click the "OK" button to close this frame. You may also close this frame by clicking the close-box in the upper left or right-hand corner of the frame (depending on your computer's operating system).

<u>**Available Context Set / Genomic Grouping Types**</u>

(1) Group genes based on intergenic distance

Annotated features are organized into non-overlapping groups based on (1) intergenic distance and (2) strandedness. An **intergenic distance threshold** field allows the user to specify a cutoff point for grouping annotated features into the same genomic grouping. In other words, the end of one annotated feature must be no further away from the start of the next annotated feature for these annotated features to be grouped into the same genomic grouping. If the "Genes must be on same strand" checkbox is checked, then genes must also be on the same strand.

This genomic grouping method is often used when organizing the genes on microbial genomes into operons.

Push the **Compute** button to sort all annotated features in all genomes in the genomic working set into the appropriate genomic groupings.   Once this computation is finished (a progress bar will appear), you may push the **add** button to load this context set into your set of available context sets.

(2) Group genes based on nucleotide range

Genomic groupings are determined by including all annotated features that are at least partially contained within the defined range of nucleotides around query matches. The range of values around query matches to take may be edited in the **nt Before** and **nt After** text fields. For this context set, it is appropriate to push the **add** button after specifying values for the number of nucleotides before and after the center of the query match to include in the context set.

(3) Group genes based on number of nearby genes

Genomic Groupings are determined by taking some number of annotated features both before and/or after all query matches. The number of features to include may be edited in the **Genes Before** and **Genes After** text fields. For this context set, it is appropriate to push the **add** button after specifying values for the number of genes before and after the query match to include in the context set.

(4) Group all genes between two queries together

All annotated features between and including two query matches are included into genomic groupings. **This genomic grouping requires that exactly two queries be provided**. Failure to do so will result in an error message. In the case that multiple instances of one or more of the individual query types exist in an annotated genome, JContextExplorer will use the pairings that result in the smallest total distance between annotated features of each type. For this context set, it is appropriate to push the **add** button once the radio button is selected.

(5) Group multiple independent queries together

Typically, multiple queries are treated as OR statements. With this context set, however, all query matches within a single genome are placed into the same genomic grouping. For example, a homology cluster search of "15; 16; 17" will result in grouping all instances of the annotated features with homology cluster numbers 15, 16, and 17 into a common gene grouping, for each genome searched. For this context set, it is appropriate to push the **add** button once the radio button is selected.

(6) Load gene groupings from file

The user may wish to define genomic groupings in one or more organisms using a method not supported by JContextExplorer (for example, as a result of an experiment). They may load these genomic groupings into JContextExplorer by creating a file called a **Context Set File**, a two-column

tab-delimited file which should contain, in **column 1**, the name of the organism, and in **column 2**, the full path to another file (an individual **context file**).

An individual **context file** should be created for each organism of interest. Each file should be a 4-column tab-delimited file, with the following information in each column:

column 1: Sequence name

column 2 : Annotated feature start position

column 3: Annotated feature stop position

column 4: Context set ID Number

Please format files carefully prior to import into JContextExplorer.

(7) Create a new context set by combining existing context sets

This feature has not yet been implemented yet. When implemented, it will attempt to allow integration /combination of multiple alternative context set creation schemes into a single method.

View Annotations

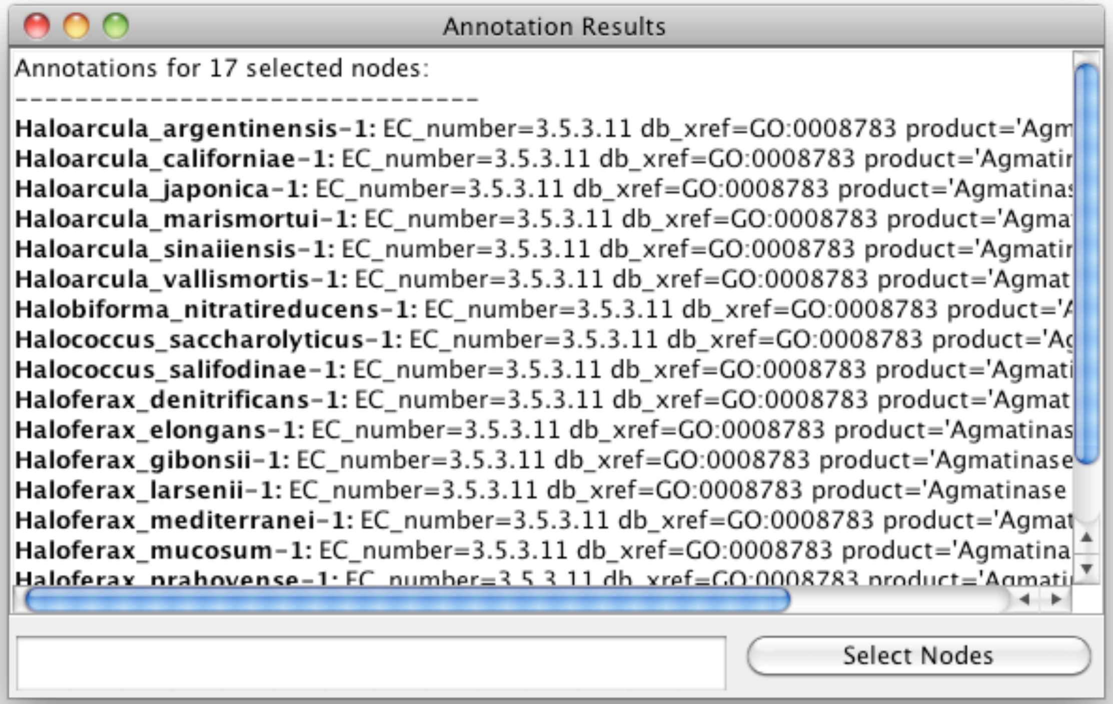

This is the “View Annotations” frame. It can be launched from within the main frame (please see **Launching Subordinate Windows and Selecting Nodes, page XXX**). The annotation frame produces a selectable text window, containing the annotation for the query match associated with each leaf in the context tree window.

The first line always lists the number of nodes selected (in this example, 17 nodes are selected). Following this, the node name is given in bold, followed by the annotation associated with the query match is given in regular text.

A search bar exists below which allows the user to type one or more node name queries (or textual fragments), and select appropriate nodes in the tree. This search bar works the same as the node selection bar in the main frame (Please see **Launching Subordinate Windows and Selecting Nodes, page XXX**).

CONTEXT TREE FRAME

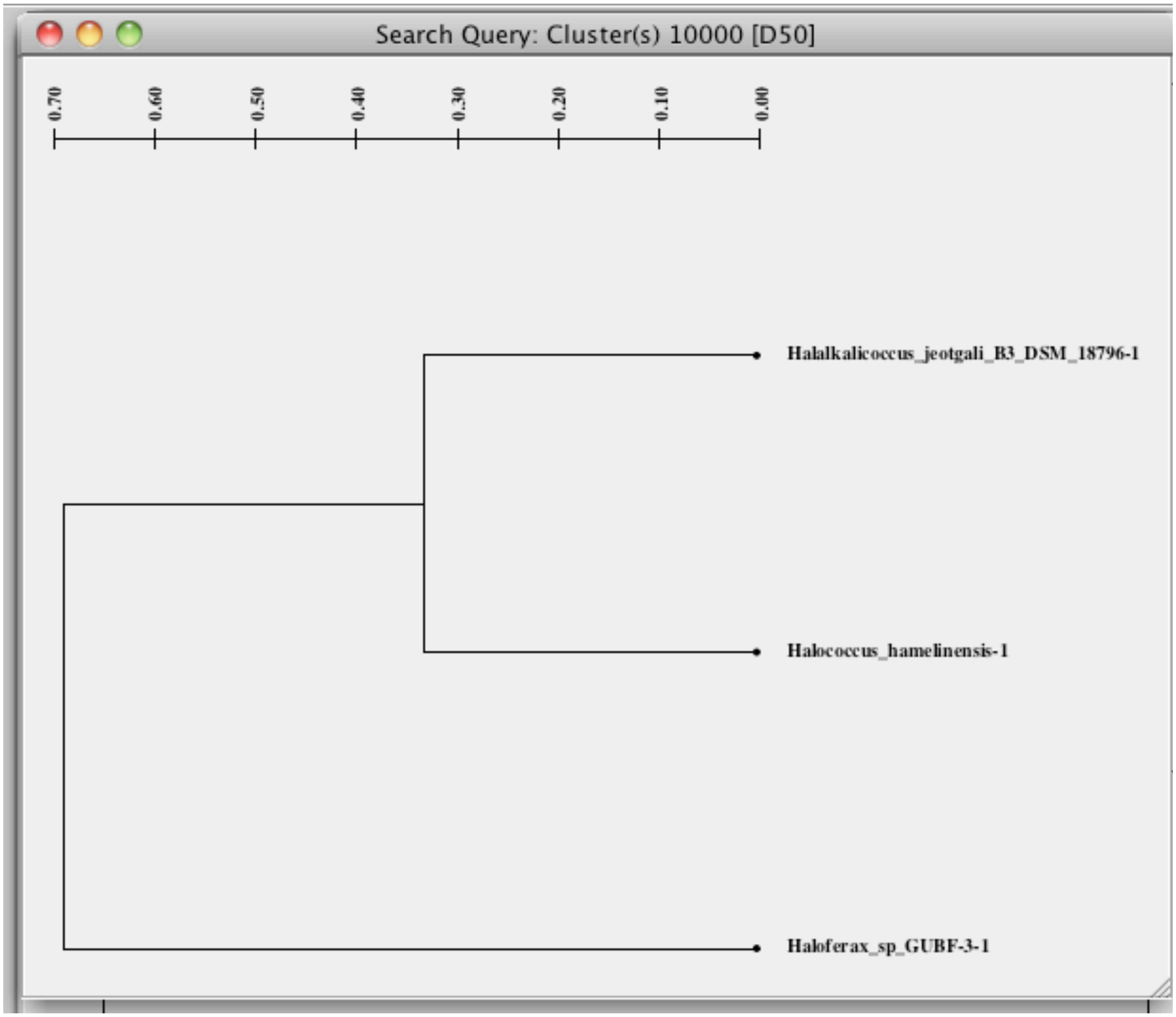

This is an example of a "Context Tree" frame. These frames appear as internal frames to the JContextExplorer main frame.

Upon performing a search using JContextExplorer, will appear in a new **Context Tree** frame as a generated context tree. As a reminder, the context tree displayed in the frame is a function of the (1) search query, (2) context set, (3) pairwise dissimilarity metric, and (4) clustering (or linkage) method. **The display and computation of the tree may be re-determined with a new context set,**

dissimilarity metric, or clustering method by changing all parameters appropriately, and clicking the "Update Tree" button in the main frame. Alternatively, changing parameters and clicking the **Submit** button in the main frame retains the old context tree, and creates a new context tree using the updated parameters.

Multiple context tree frames may be managed simultaneously – they may be moved, re-sized, minimized, maximized, closed and restored. Multiple investigations may occur simultaneously through minimizing and restoring individual context tree frames.

Tree frames are interactive both by (1) Left Click and (2) Right Click. Left clicking on a frame selects leaves for annotation / context viewing, while Right clicking launches a pop-up menu showing several additional available options. **A red rectangle will appear around Selected leaves.**

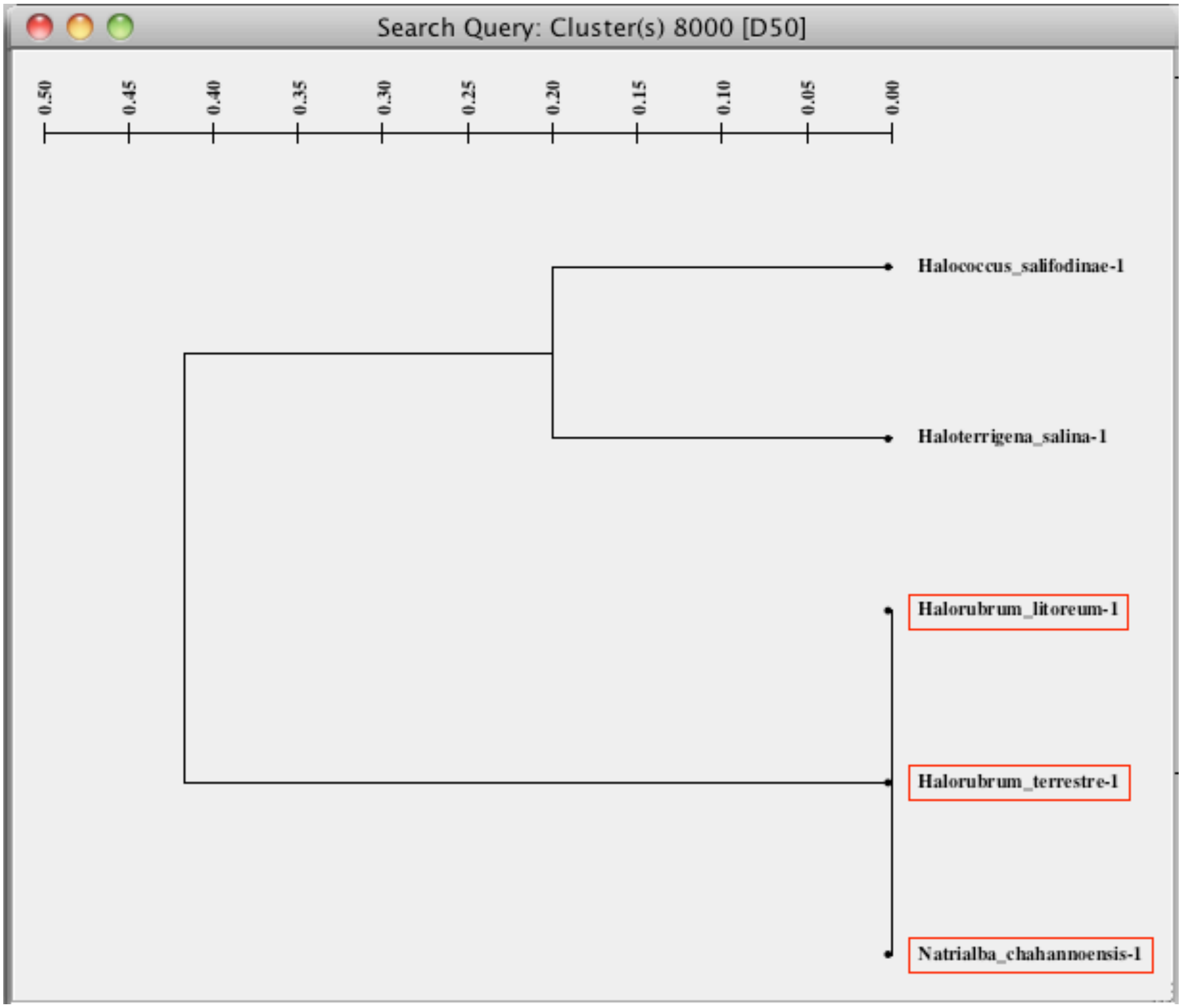

Example: the 3 nodes on the bottom of this tree are selected, while the two nodes on the top are unselected.

Left Click Options

Clicking on an individual **leaf node name** will select it. Clicking on a different leaf node name will de-select the previously selected leaf node, and select the new leaf node name.

Holding down the **CTRL** key while clicking on a leaf node will select that leaf node (if it unselected) or deselect that leaf node (if it is selected), **without changing the selection profile of the other leaf nodes**.

Holding down the **SHIFT** key while clicking on a leaf node will select every leaf node between the currently selected leaf node and the closest previously selected leaf node.

Right Click Options

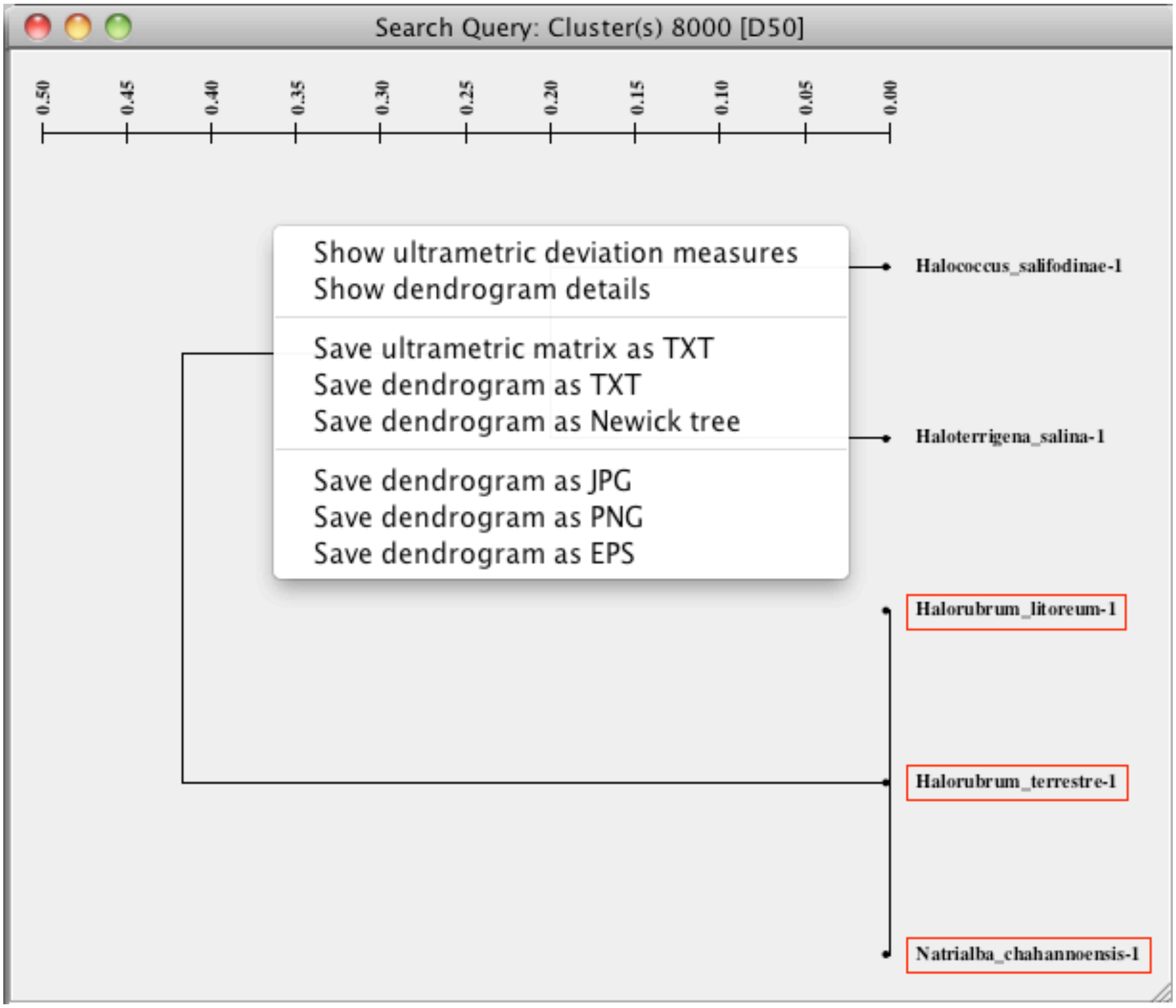

Right-clicking anyway on the frame will bring up the pop-up menu shown in the figure above.

These options are borrowed from the original MultiDendrograms software package:

Gomez, S., Fernandez, A., Montiel, J., & Torres, D. (n.d.). Solving Non-Uniqueness in Agglomerative Hierarchical Clustering Using Multidendrograms. *Journal of Classification*, *65*, 43-65. doi:10.1007/s00357-008-

A complete user manual is available at http://deim.urv.cat/~sgomez/multidendrograms.php#description

Please refer to this documentation for more information.

MULTI-GENOME BROWSER / CONTEXT VIEWER

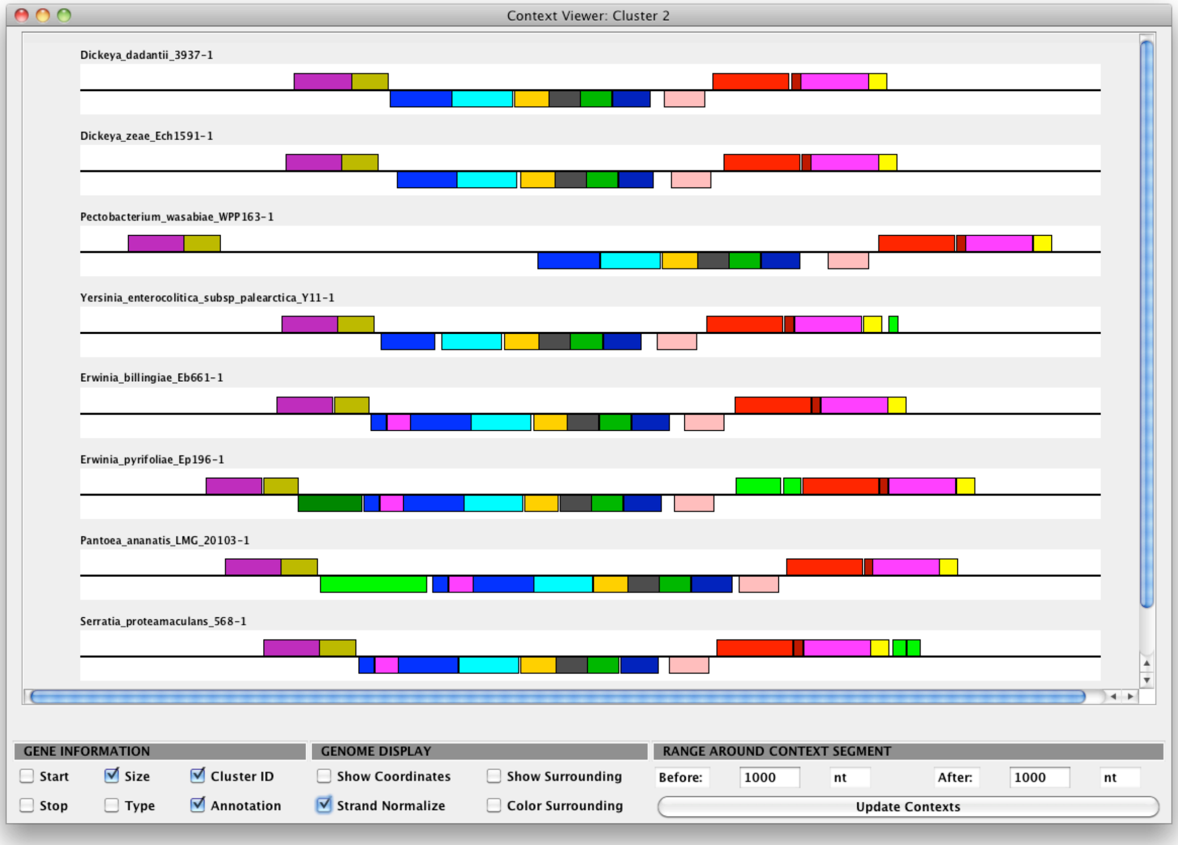

This is an example of the Context Viewer /Multi-Genome browser frame. This frame may be launched from the main frame (see **Launching Subordinate Windows and Selecting Nodes, page 23**). When launching this frame, a set of leaves on a context tree must also be selected.

The purpose of this frame is to visualize the genomic groupings associated with the leaves on the active context tree. Annotated features are rendered as colored rectangles (**colored according to common homology cluster ID number or common annotation, depending on how the context tree was generated**) resting either above (for plus-stranded features) or below (for minus-stranded features) a single black line, in the order they appear in the associated annotated genome.

The associated node name is printed above and to the left of each rendered genomic segment.

The ContextViewer is an active frame. Left clicking, right clicking, and center clicking on individual genes and parts of the frame do different things. Individual **Option sub-panes** in the bottom left, bottom center, and bottom right also have interactive effects.

__Gene Information__

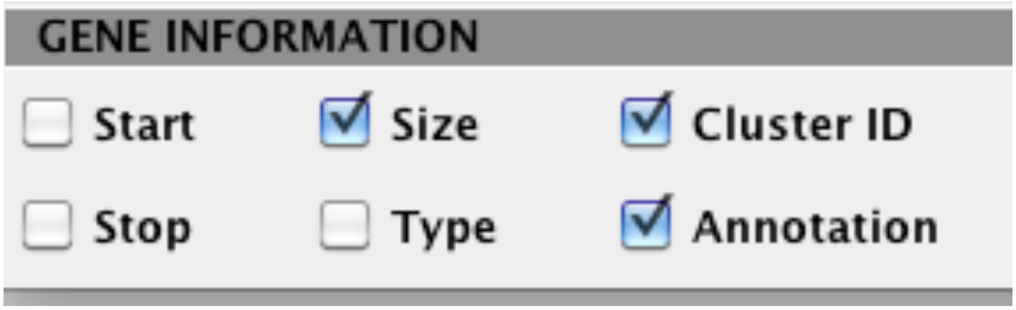

This is the **Gene Information sub-pane**. These check boxes describe which biological information should be displayed upon **left clicking** on an individual annotated feature in the ContextViewer frame.

__Genome Display__

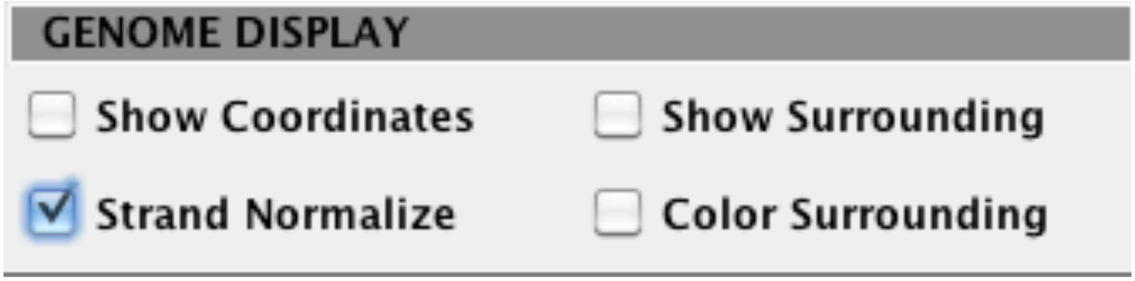

This is the **Genome Display sub-pane**. These check boxes describe how whole genomic segments should be rendered in the above ContextViewer pane.

if **Show Coordinates** is selected, numerical values will appear below individual rendered genomic segments displaying coordinates every 1000 nt or so. The name of the sequence will also appear in the upper-left hand corner, and a small triangular **flag** will appear in the upper left-hand corner of each genomic segment,

pointing in increasing order. If this flag is black (and pointing to the right), the sequences are increasing left to right, if the flag is red (and pointing to the left), the sequence is displayed in reverse complement, and so is increasing right to left.

If **Show Surrounding** is checked, annotated features that are not a member of the genomic grouping associated with the genomic segment displayed will also be displayed. These features may either be displayed as colored or gray, depending whether or not **Color Surrounding** is checked or unchecked.

If **Color Surrounding** is checked, annotated features will be colored according to common homology group ID or common annotation, just as the genomic groupings are colored. If **Show Surrounding** is unchecked, this option has no effect.

If **Strand Normalize** is checked, individual genomic segments may be displayed in sequence reverse complement so that query matches are on the forward strand. If the genomic segment is already oriented such that query matches are displayed on the forward strand, this option has no effect.

<u>Range to Display</u>

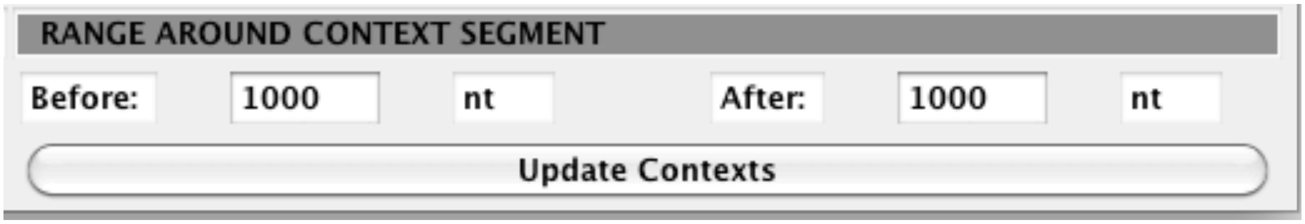

This is the **Range to Display sub-pane**. This controls how much of the surrounding genomic region should be displayed along with individual genomic groupings. Changing values in the “Before” and “After” text fields, and clicking the **Update Contexts** text field will re-render all genomic segments in the range to display sub-pane.

The **Update Contexts** button is also linked to the leaves selected on the associated **Context Tree** for the rendered contexts: **You may change the**

rendered contexts by changing the leaves selected in the context tree frame and pushing the Update Contexts button.

<u>Left Clicking</u>

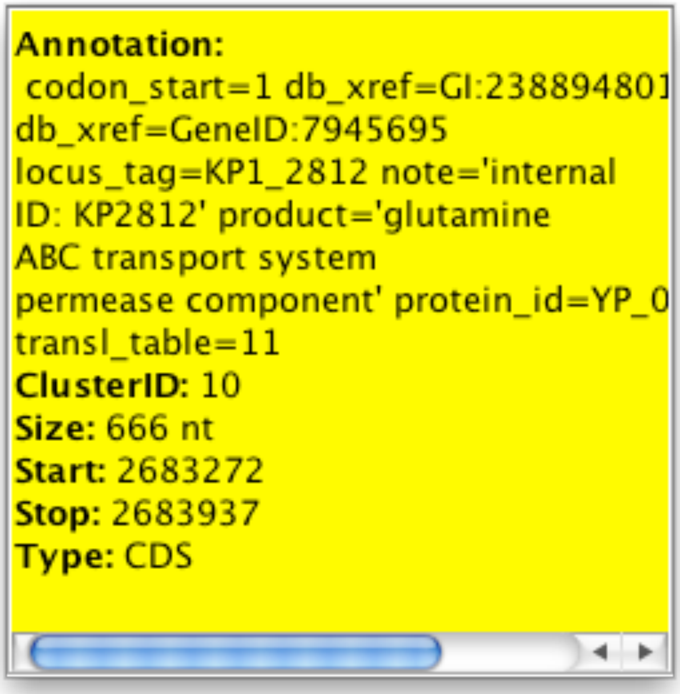

This is the **gene information sub-frame. Left clicking** on an individual annotated feature results in a small, yellow box appearing at the point of clicking, displaying biological information about the annotated feature clicked.

Left click on another part of the frame that does not contain an annotated feature to make this frame disappear; left click on a different annotated feature to display biological information for that annotated feature.

Right Clicking

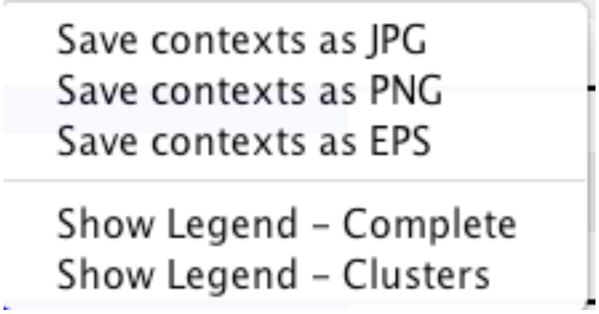

Right clicking anywhere on the frame opens the pop-up menu displayed above. left clicking away causes this popup menu to disappear.

Selecting any of the **image export** options will open a file dialog allowing for image export. In the image export, only the rendered genomic contexts will appear, and they will always appear exactly as they do on screen. Selecting any of the **Show Legend** options will launch the **Gene Color Legend** frame (please see **Gene Color Legend, page XXX**).

Middle Clicking

Middle clicking on a particular annotated feature will select all other annotated features with the same homology cluster or annotation (depending on the initial search type).

You may hold down the **CTRL** or **SHIFT** key while middle clicking, which will allow for selection of multiple annotated feature groups. If you have the **Gene Color Legend** frame open, then the entry associated with this annotated feature will also appear selected (surrounded by a thin, red rectangle).

GENE COLOR LEGEND

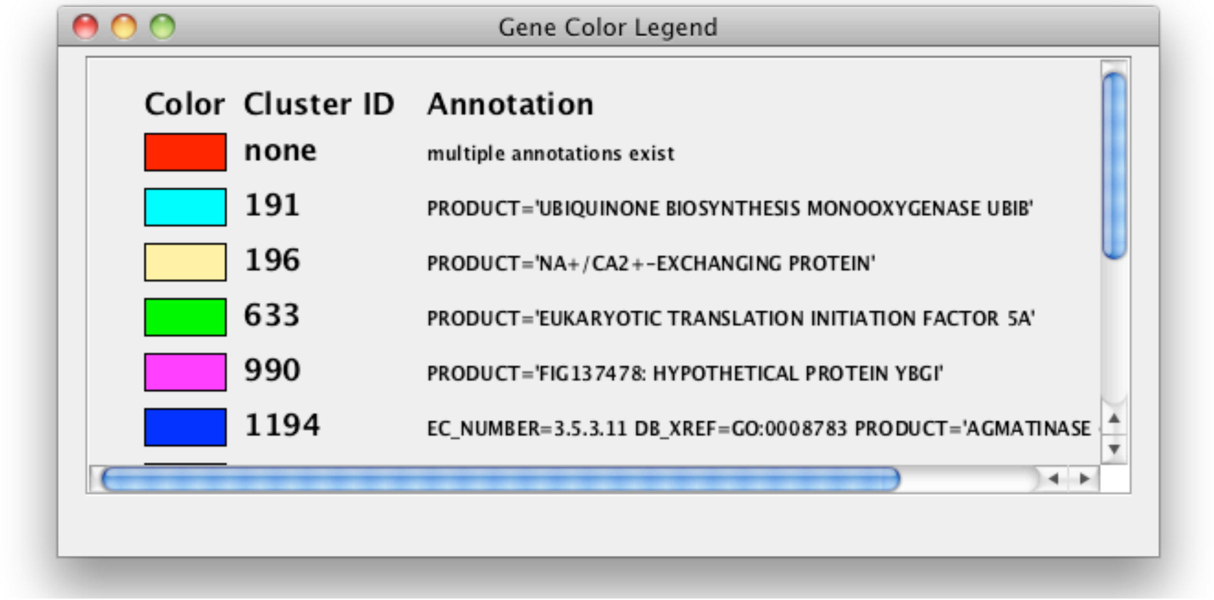

This is the Gene Color Legend frame. It contains the mapping between colors, cluster ID, and annotations associated with its parent **ContextViewer** frame.

The Gene Color Legend is an active frame. You may **Left Click / Middle Click** or **Right Click**. If you Left or Middle click, you will select the associated color – clusterID – annotation relationship in the frame, as well as in the parent ContextViewer window.

Holding down the **CTRL** key while clicking on a color – clusterID – annotation mapping will select that mapping (if it unselected) or deselect that mapping (if it is selected), **without changing the selection profile of the other mappings**.

Holding down the **SHIFT** key while clicking on a leaf node will select every mapping between the currently selected mapping and the closest previously selected mapping.

Selections in this frame will appear in the parent **ContextViewer** frame.

If you right click anywhere on the frame, you will open a pop-up menu allowing for various figure export options (as **.jpg, .png**, or **.eps** files).

CHAPTER 4: ADDITIONAL RESOURCES

TUTORIAL I: DETERMINING HPXW FROM GGT IN 22 ALPHA AND GAMMA-PROTEOBACTERIA

In this tutorial, we will recapitulate the analysis described in the JContextExplorer manuscript.

(1) Retrieve Information

Please download the associated biological information for 22 alpha and gamma proteobacteria from the Facciotti lab website:

http://www.bme.ucdavis.edu/facciotti/resources_data/software/

Please click the hyperlink entitled "AlphaAndGammaProteobacteria", and extract the contents of the directory. The resulting extracted directory should contain (1) a file titled "HomologyCluster.txt" (2) a directory titled "Annotations" and (3) a file titled "AlphaAndGammaProteobacteria.nwk".

(2) Load JContextExplorer with tutorial dataset

(A) Either by downloading the zipped JContextExplorer directory and launching locally, or using the Java WebStart on the Facciotti Lab Website, launch JContextExplorer.

(B) Select the newly downloaded / extract **AlphaAndGammaProteobacteria/Annotations** directory for genomic working set.

(C) When the intermediate GFF-checking frame appears, click the **"Proceed to GFF Import with these type-processing settings"** button.

(D) Once this has finished loading, load the homology clusters in the newly extracted directory: **AlphaAndGammaProteobacteria/HomologyClusters.txt**

(E) Once this has loaded, click the submit button, which should launch the JContextExplorer main frame.

(3) Define the D75 Context Set

(A) In the upper left-hand corner of the main frame, below the banner that says **Select Context Set**, click the **Add/Remove** button. When you have done so, you will see a window that looks like the Add/Remove Context Sets window (see **page 24**).

(B) In the text field to the right of **Enter Name:** , type D75

(C) Select the radio button "Group genes based on intergenic distance"

(D) In the text field directly under this radio button, change "20" to "75". Leave the **Genes must be on the same strand** option checked. Then, click the **compute** button next to this field. This will take a few seconds to compute.

(E) Once it has finished, find the "Add" button ¾ of the way down the frame, and click this button.

Note that in the Context Set drop-down menu (near the bottom of this frame), you will now see "D75" in the list.

(F) Click the **OK** button at the bottom of the frame.

(4) Set appropriate parameters + conduct search

The following 6 steps are displayed and described below.

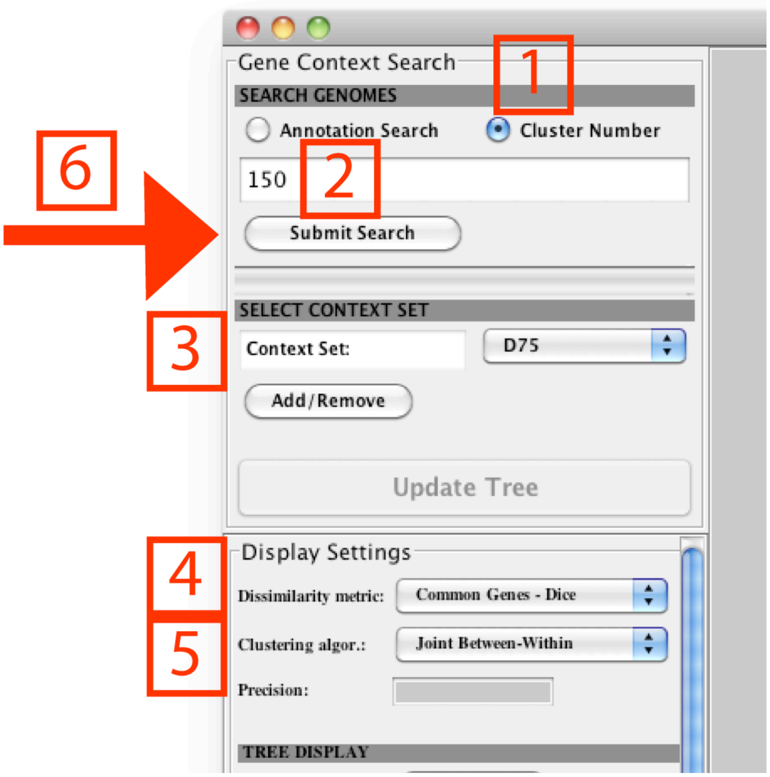

Returning to the main frame, in the upper left-hand corner, in the "Search Genomes" sub-panel, you should see two radio buttons, one titled "Annotation Search" and the other titled "Cluster Number". **Make sure "Cluster Number"** is selected **(1)**. In the text field below, type the number "**150**" **(2)**. Below this, you should see the banner titled **Context Set**. From the drop-down menu, select the newly created context set, **D75 (3).** Directly below this panel, you should see another panel titled "Display Settings". In the drop-down menu associated with Dissimilarity Metric, select "**Common Genes – Dice**" **(4).** Directly below, in the drop-down menu associated with Clustering Algorithm, select "**Joint Between-Within**" **(5).** Finally, return to the "Search Genomes" sub-panel and push the "Submit Search" button **(6).**

(5) Select portion of Tree for Context Viewing

The following 5 steps are displayed and described below.

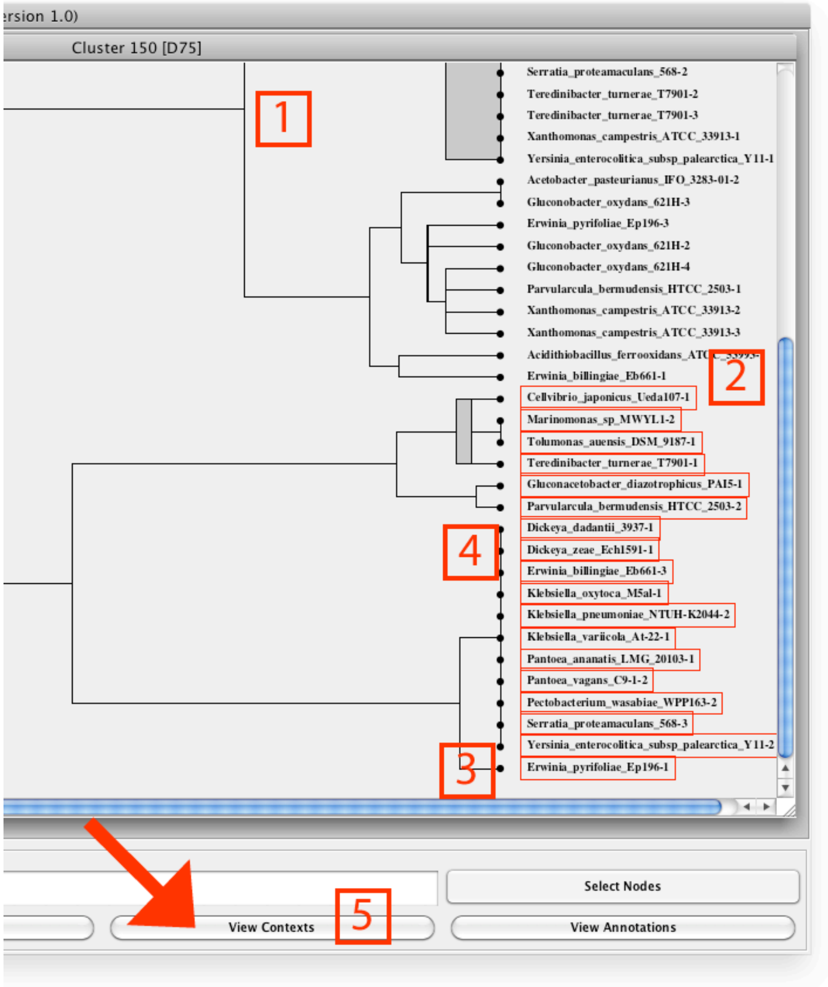

JContextExplorer now searches through all genomes for genes in homology cluster 150, returns all associated gene groupings, and renders a tree **(1)** comparing all gene groupings in all organisms containing a gene in homology cluster 150. To view the actual contexts, scroll down on the internal child window as far as you can go and right-click on the node labeled “Cellvibrio_japonicus_Ueda107-1” **(2).** Hold down the shift key, and right-click on the node “Erwinia_pyrifoliae_Ep196-1” **(3).** This will select all intermediate nodes **(4).** Finally, click on the “View Contexts” button **(5).** This will bring up the multi-genome browser context viewer window.

TUTORIAL 2: COMPARING A JCONTEXTEXPLORER-GENERATED CONTEXT TREE TO A PHYLOGENETIC TREE

This tutorial is an extension of the previous tutorial. Please perform all steps in tutorial 1 to the point where you have generated a context tree for homology cluster 150 in the D75 context set, using the Joint-Between Within clustering algorithm and Common Genes – Dice Dissimilarity measure.

(1) Export the context tree.

With the previously generated context tree in the active window, right click anywhere within the context tree frame. A pop-up menu should appear. Select the 5th option from the drop-down list – **save dendrogram as Newick tree**. Save this tree somewhere on your file system.

(2) Launch TreeJuxtaposer

Open an Internet browser and navigate to the TreeJuxtaposer downloads page:

http://olduvai.sourceforge.net/tj/download.shtml

At the top of the page, you should see a link to launch the WebStart. Click this button to launch the WebStart.

(3) Load Context Tree and pre-computed Phylogenetic Tree

If you have not downloaded and extracted the contents of the zipped AlphaAndGammaProteobacteria package from the Facciotti lab website, please do so. In the extracted package, you should discover a file titled **AlphaAndGammaProteobacteria/AlphaAndGammaProteobacteria.nwk**

This is a whole-genome phylogenetic tree. In the TreeJuxtaposer package, open this file using **file -> open**. This will launch the phylogenetic tree. While this file is loaded in the window, open the JContextExplorer-generated context tree in the same window, again using **file -> open**.

(4) Explore the features of TreeJuxtaposer

TreeJuxtaposer is well documented. Please see the documentation page:

http://olduvai.sourceforge.net/tj/documentation.shtml

VIDEO TUTORIALS

Several video tutorials are publically available on youtube. To visit the video page, please navigate to

http://www.youtube.com/user/jcontextexplorer?feature=results_main

There are currently 5 video tutorials available on this youtube movie channel.

The first three videos provide a brief introduction to JContextExplorer, demonstrate how to retrieve the program, and demonstrate using JContextExplorer.

Video 01: Introduction

http://www.youtube.com/watch?v=SJ1wcsnErsg&feature=plcp

Video 02: Retrieval

http://www.youtube.com/watch?v=ZbWAd-oXHu4&feature=plcp

Video 03: Usage

http://www.youtube.com/watch?v=zlt2qTeVe7o&feature=plcp

These three videos are available in a single video playlist:

http://www.youtube.com/watch?v=SJ1wcsnErsg&list=PLPCF4X54wfPnrlPCqZ2gEb9aaQrLSyE6Y&feature=plcp

The next two videos highlight recent updates in features, showing the version current as of November 17, 2012, and perform many of the steps of tutorials 1 and 2.

Video 04: Updates November 17, 2012

http://www.youtube.com/watch?v=sJNdsGGma-8&feature=plcp

Video 05: Export a Context Tree

http://www.youtube.com/watch?v=EehARCQ1jTc&feature=plcp

These two videos are available in a single video playlist:

http://www.youtube.com/watch?v=sJNdsGGma-8&list=PLPCF4X54wfPlvaufZd_n7pO799m5klLXw&feature=plcp

Author Contact Information

The chief author of this manual and software is Phillip Seitzer. He can be reached by email at pmseitzer@ucdavis.edu

Phillip Seitzer is a member of the Facciotti lab at the University of California at Davis:

http://www.bme.ucdavis.edu/facciotti/

The source code for JContextExplorer is hosted on GitHub:

https://github.com/PMSeitzer/JContextExplorer

Please do not hesitate to contact the author with questions, comments, bug reports, feature requests, and more.
