# Supplementary material for: JContextExplorer: a tree-based approach to facilitate cross-species genomic context comparison
Source: BMC Bioinformatics. 2013 Jan 16;14:18. doi: 10.1186/1471-2105-14-18 (PMC3560190; doi:10.1186/1471-2105-14-18)
Supplement: Additional file 2 — Supplementary Figures and Methods related to separating hpxW from ggt in 22 alpha and gamma proteobacteria. Visualization of hpxW contexts, comparison of whole-species phylogeny to hpxW sub-portion of JContextExplorer-generated context tree, and detailed description of methods associated with hpxW/ggt analysis. [file 1471-2105-14-18-S2.doc]

**Supplementary Information**

Table of Contents

S1: Supplementary Figure 1 2

hpxW context from the hpxW sub-tree 2

S2: Supplementary Figure 2 4

Comparison of Species Phylogeny and hpxW sub-tree 4

S3: hpxW / ggt Analysis - Methods 6

Generation of genomic working set and homology clusters 6

Generation of context tree 6

Generation of phylogenetic tree 7

Supplementary References 8


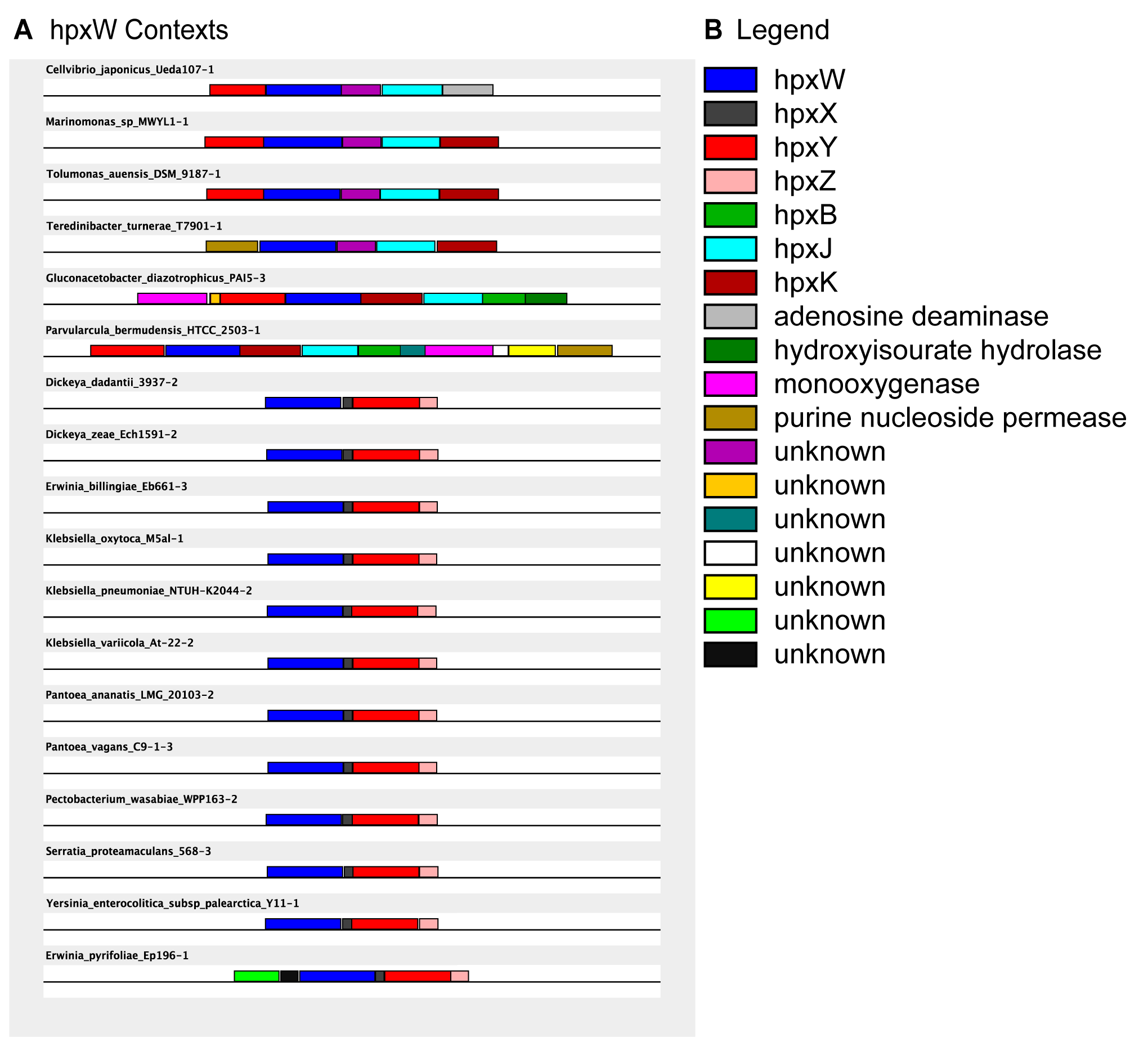


(S1) Supplementary Figure 1: hpxW contexts from the hpxW sub-tree

(A) Gene groupings from the hpxW sub-tree of the ggt/hpxW context tree (Supp Fig. 1) are rendered here using JConextExplorer’s multi-genome browser context viewer tool. Biological hypotheses are easy to glean using this tool, which encourages “by-eye” parsing of differences between genomic groupings across species. All gamma-proteobacteria in the family Enterobacteriaceae contained genes hpxW, hpxX, hpxY, and hpxZ in their context sets. Interestingly, two additional genes of unknown function grouped upstream of the hpxW gene in the enterobacteriacial species *E. pyrifoliae Ep196.* One might hypothesize that these genes represent single-gene insertions. We note that in several species, we observe the genes hpxB, hpxJ, and hpxK, which have been implicated in purine catabolism [38]. We also find several other genes potentially functionally related to purine catabolism, such as monooxygenase (found in the alpha-proteobacteria *P. bermudensis* and *G. diazotrophicus*) and purine nucleoside permease (found in the alpha-proteobacterium *P. bermudensis* and the non-enterobacteriaceae gamma-proteobacterium *T. turnerae*). (B) Colors and associated annotations are provided in this illustration, and are also readily accessible in the JContextExplorer tool. Genes annotated as hpx* were assigned based on expert biological knowledge; all others were taken from the NCBI refSeq genome repository [S1].


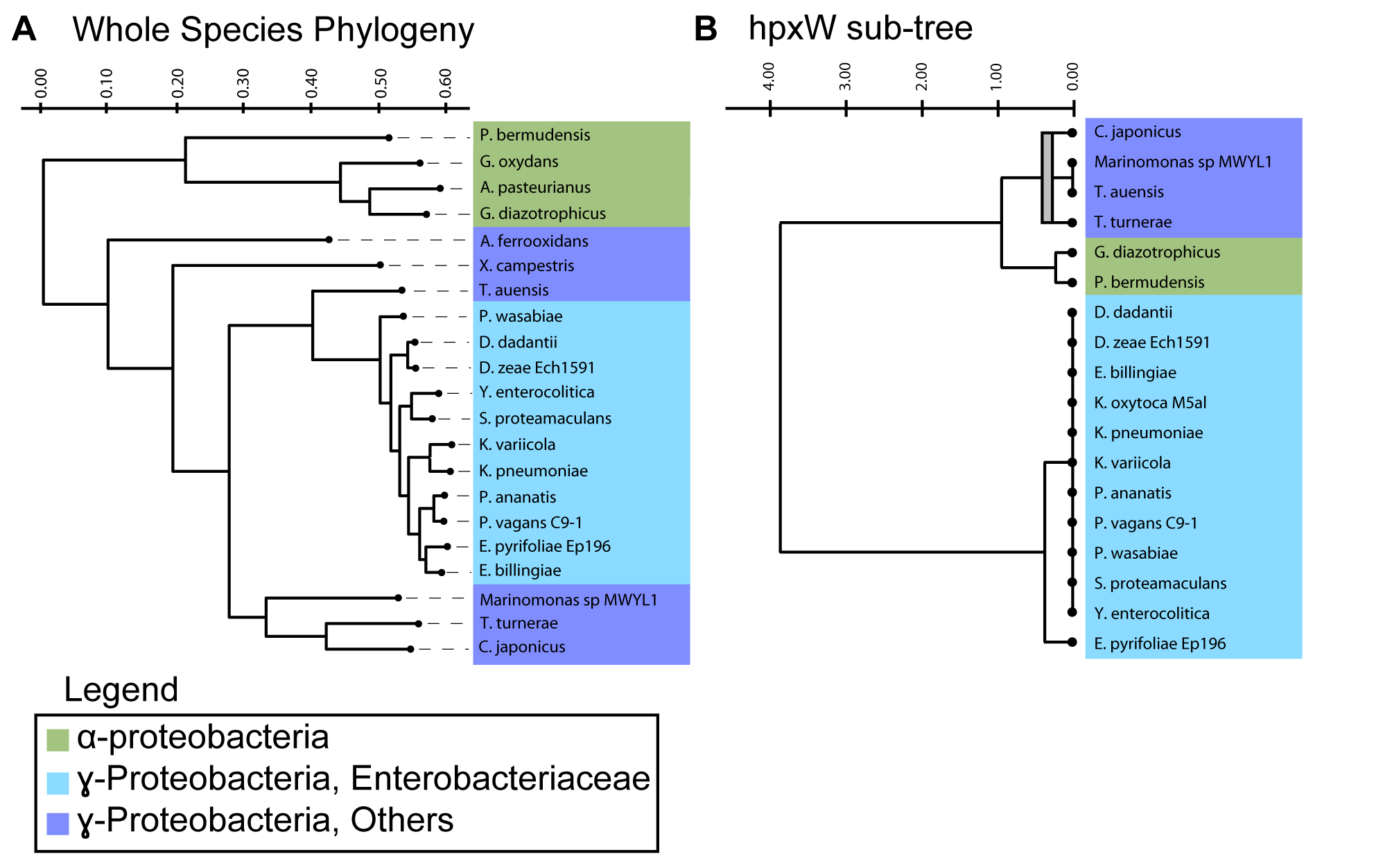


(S2) Supplementary Figure 2: Comparison of Species Phylogeny and hpxW sub-tree

(A) A phylogenetic tree of the 22 alpha and gamma – proteobacteria was created using Phylosift [S2] and FastTree [S3]. Species were found to group as expected according to their taxonomic classification: all gamma-proteobacteria in the family enterobacteriaceae (light blue) grouped together closely, and all gamma (light blue and purple) and alpha (green) proteobacteria grouped into separate branches. The portion of the hpxW/ggt context tree (Fig. 3) corresponding to the hpxW genes is reproduced here, and species are highlighted according to their taxonomic classification (B). Comparing the phylogenetic distribution of species with the distribution of species in the hpxW sub-tree, we observe that organisms cluster together at the lowest level according to their phylogeny (all gamma-proteobacteria in the family enterobacteriaceae group together, all non-enterobacteriaceae gamma-proteobacteria group together, and all alpha-proteobacteria group together), however the tree does not recapitulate the phylogenetic topology one level up: the alpha-proteobacteria cluster with the non-Enterobacteriaceae gamma-proteobacteria, and this group then clusters with the enterobacteriaceae gamma-proteobacteria.

(S3) hpxW / ggt Analysis - Methods

Generation of genomic working set and homology clusters

The complete genomes of 22 alpha and gamma proteobacterial species (annotated genomes available in Additional File 2) were downloaded from the NCBI website using the RefSeq tool [S1]. All coding regions in all genomes were translated, and a BLASTp all-v-all analysis was undertaken with an E-value cutoff of 10^-5^ [33]. All annotated coding regions were assembled into homology clusters by processing the BLAST results with tribe-MCL [34] with an inflation parameter of 1.4. These clusters were re-formatted into a tab-delimited file format appropriate for import by JContextExplorer.

Generation of context tree

The genomes of all 22 alpha and gamma-proteobacterial species, with corresponding homology clusters were loaded into JContextExplorer. Using JContextExplorer’s computational context set prediction tool, a “D75” context set was created that grouped all same-species genes on the same strand within 75 nucleotides from each other into common gene groupings. An annotation search was undertaken for “glutamyltranspeptidase”, which returned several results. Manual curation of these results using JContextExplorer’s multi-genome browser context viewer tool suggested that the homology cluster with ID number 150 contained all ggt and hpxW genes.

A cluster search was carried out on the loaded genomic woroking set, homology clusters, and D75 context set for cluster ID 150, which returned 51 results. These results were assembled into a tree using the “Common Genes – Dice” dissimilarity metric and “Joint Between-Within” linkage function (tree is shown in **Fig 3)**.

Generation of Phylogenetic Tree

The whole genome sequences of all species were analyzed using the PhyloSift program [S2]. Protein alignments were concatenated and analyzed with FastTree [S3], and re-rooted to the midpoint using MATLAB’s bioinformatics toolbox phylogenetic tree analysis feature [S4].

**Supplementary References**

S1. Pruitt, K. D., Tatusova, T., & Maglott, D. R. **NCBI reference sequences (RefSeq): a curated non-redundant sequence database of genomes, transcripts and proteins.** *Nucleic acids research* 2007, **35**:D61-5.

S2. PA. Darling, H. Bik, G. Jospin, J. A. Eisen. Manuscript in preparation. Retrieved from phylosift.wordpress.com

S3. Price, M. N., Dehal, P. S., & Arkin, A. P. **FastTree 2--approximately maximum-likelihood trees for large alignments.** *PloS one* 2010, **5**:e9490.

S4. Schmidt, H., & Jirstrand, M. **Systems Biology Toolbox for MATLAB: a computational platform for research in systems biology.** *Bioinformatics (Oxford, England)* 2006, **22**:514-5.
